# Multi‐State Probabilistic Computing Using Floating‐Body MOSFETs Based on the Potts Model for Solving Complex Combinatorial Optimization Problems

**DOI:** 10.1002/adma.202516797

**Published:** 2026-02-17

**Authors:** Sunwoo Cheong, Soo Hyung Lee, Janguk Han, Jun‐Young Park, Dong Hoon Shin, Yoon Ho Jang, Sung Keun Shim, Sungho Kim, Cheol Seong Hwang, Joon‐Kyu Han

**Affiliations:** ^1^ College of Engineering, Department of Materials Science and Engineering and Inter‐university Semiconductor Research Center Seoul National University Seoul Republic of Korea

**Keywords:** annealing, combinatorial optimization, floating body MOSFET, multi‐state, probabilistic computing

## Abstract

Probabilistic computing has gained attention for solving combinatorial optimization problems (COPs), mainly using the Ising model, which may not be suitable for complex COPs. Instead, this work proposes a multi‐state probabilistic computing system based on the Potts model using stochastic threshold switching floating‐body metal‐oxide‐semiconductor field‐effect transistors (FB‐MOSFETs) as the multi‐state probabilistic bits (p‐bits) to solve challenging COPs. The system employs drain voltage sharing and a one‐hot sampling method to achieve controllable probabilistic behavior and scalable annealing. Experimental validations on spin glass and max‐4‐cut problems demonstrate that the system efficiently samples a tunable Boltzmann distribution while converging faster than traditional methods. Comparative analyses further highlight superior energy efficiency and decreased time‐to‐solution, underscoring the potential of multi‐state probabilistic computing for large‐scale, complex COPs using only MOSFET devices.

## Graphical abstract

This work introduces a multi‐state probabilistic computing system based on Potts‐model p‐bits using stochastic switching floating body metal oxide semiconductor field effect transistors (FB‐MOSFETs). By employing drain‐voltage sharing and one‐hot sampling, the system achieves controllable probabilistic behavior. Experiments on spin‐glass and max‐4‐cut problems demonstrate faster convergence and improved energy efficiency, highlighting its suitability for complex combinatorial optimization problems.

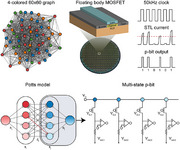

## 1 Introduction

Combinatorial optimization problems (COPs) are fundamental in various domains, including scheduling, circuit design, network design, and logistics [1, 2, 3, 4]. These problems require finding an optimal solution within a finite but exponentially large solution space, leading to the well‐known challenge of a combinatorial explosion. Addressing these challenges has been a key focus in optimization research, leading to the development of numerous algorithmic and hardware‐based approaches [5, 6, 7, 8, 9, 10, 11, 12].

Probabilistic computing using probabilistic bits (p‐bits) has recently emerged as a promising approach for efficiently solving COPs. COPs can be formulated as energy‐based models, such as the Ising model, where the problem is mapped onto a system of binary spin variables with interactions that define an energy landscape [13, 14]. The objective is to randomly sample the states from the systems that follow Boltzmann distributions and reach the global minimum of this energy landscape, corresponding to an optimal solution. The Potts model generalizes the Ising model by allowing each variable to take on one of *q* discrete states rather than being restricted to binary values. This property enables a more natural representation of complex optimization problems and phase transitions in statistical physics [15, 16]. The energy (that is, Hamiltonian) of the Potts model is expressed as Equation (1):

(1) $$E=-\left(\sum_{i,j}J_{ij}{\vec{s}}_{i}\cdot {\vec{s}}_{j}+\sum_{i}{\vec{h}}_{i}\cdot {\vec{s}}_{i}\right)$$

where ${\vec{s}}_{i}$ and ${\vec{s}}_{j}$ represent the states of neighboring nodes, *J_ij_* represents the coupling strength between *i* and *j*, and ${\vec{h}}_{i}$ is the external field acting on site *i*. The energy contribution of each state can be analyzed through Δ*E*, which is the energy difference between the previous and current states. The feedback to the system is calculated using various algorithms [5, 12, 17].

Optimization is performed by iteratively updating the node states to minimize the total energy. However, due to the highly complex energy landscape of COPs, deterministic methods often become trapped in local minima. In contrast, probabilistic computing introduces stochastic fluctuations that enable the system to escape local minima, improving the likelihood of finding a global optimum. By incorporating annealing techniques, probabilistic systems can explore a wider configuration space before settling into an optimal solution. This approach has been widely employed for solving optimization problems and modeling phase transitions in complex systems [7, 18, 19, 20].

Most previous studies on probabilistic computing have focused on the Ising model, applying it to COPs such as max‐cut, the traveling salesperson problem, and integer factorization by searching for the energy minimum [21, 22, 23, 24, 25, 26, 27, 28]. The Ising model is suitable for problems requiring binary decision variables, but more complex problems, such as the max‐K‐cut problem, require *K* discrete states per site or node [13]. The conventional Ising‐based hardware approach requires problem transformation via graph duplication for such cases, significantly increasing computational complexity [13]. Probabilistic computing with the Potts model offers a direct and more efficient solution. It inherently supports multi‐state variables without requiring additional transformations.

Although previous research has explored both Ising and Potts models [12, 29], studies on the Potts model are still limited. Specifically, existing works typically address only parts of the required system, either relying on simulations or on non‐fabricated platforms, or using fabricated devices without a large‐scale array and optimization demonstration. Furthermore, implementing practical probabilistic computing hardware requires a highly scalable system based on the well‐established complementary metal‐oxide‐semiconductor (CMOS) platform. Since the first hardware implementation of p‐bits using magnetic tunnel junctions was reported [23], various emerging stochastic devices, such as resistive memories [30, 31] and oscillators [32, 33, 34], have provided a versatile platform for energy‐based hardware. However, their two‐terminal configurations offered only limited circuit configurations. Also, integrating these devices into a mature CMOS fabrication process still incurs extra costs for contamination control and requires further process development. In contrast, probabilistic computing based solely on metal‐oxide‐semiconductor field‐effect transistor (MOSFET) with a silicon channel is more practical. However, conventional deterministic CMOS circuits lack intrinsic randomness.

This work presents a fully CMOS‐compatible multi‐state probabilistic computing system based on the Potts model, utilizing floating‐body MOSFETs (FB‐MOSFETs). The proposed system achieves controlled stochasticity by intentionally destabilizing the floating body potential, enabling probabilistic behavior in a conventionally deterministic CMOS device. A multi‐state p‐bit unit is realized by sharing a common drain voltage (*V*_D_) and adopting a one‐hot sampling method, ensuring robust multi‐state operations. By dynamically tuning the gate voltage (*V*_G_), the probability distribution across multiple states can be precisely controlled, providing high flexibility in annealing‐based optimization.

Its capability was experimentally validated to generate Boltzmann‐distributed outputs, which are crucial for probabilistic sampling and optimization tasks, demonstrating the feasibility and effectiveness of this approach. Furthermore, utilizing intrinsic asynchronicity in the one‐hot sampling method allows scalable annealing schedules and efficient parallel updates, significantly limiting the proportion of nodes flipped in each iteration [25, 35, 36]. Experimental demonstrations on spin glass and max‐4‐cut problems using a custom hardware platform highlight the effectiveness. Furthermore, the optimization results are compared to software optimization algorithms across various metrics.

## 2 Results

### 2.1 Fundamental Electrical Characteristics and p‐Bit Operation of the FB‐MOSFET

**FIGURE 1:**
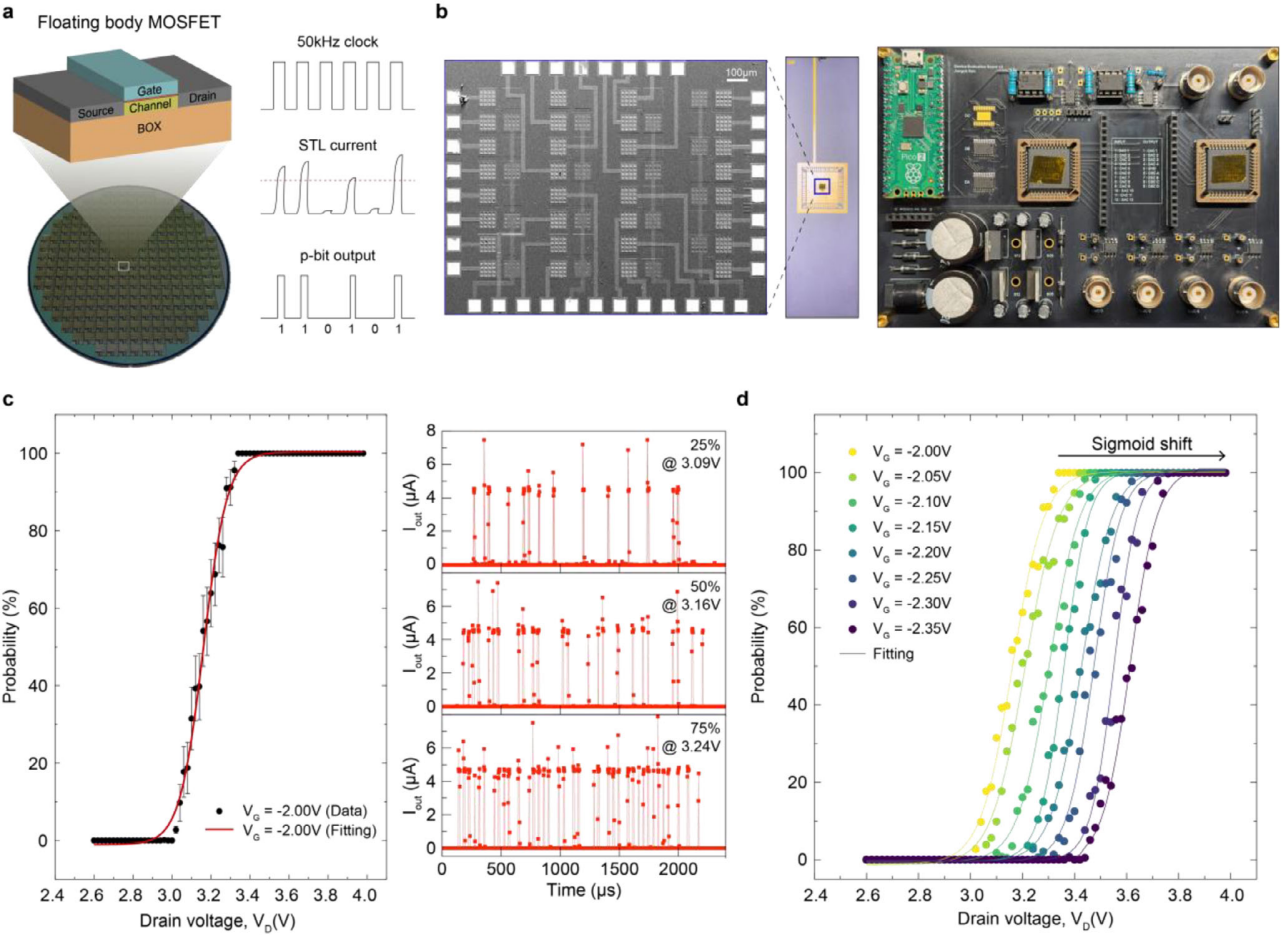
P‐bit operation based on FB‐MOSFET. (a) Schematic of a FB‐MOSFET and fundamental binary p‐bit behaviors. FB‐MOSFET was fabricated on an 8‐inch wafer using CMOS technology. (b) Experimental setup for the proposed multi‐state p‐bit system. A top‐view SEM image of drain‐shared four FB‐MOSFETs. Four p‐bit nodes were packaged through wire bonding (left). A custom board with the MCU supported the sampling of p‐bits (right). (c) Sigmoidal probability of single transistor latch (*P*_STL_) according to drain voltage (*V*_D_), which is the fundamental binary p‐bit operation (left). The cycle‐to‐cycle variation over 10 epochs is expressed as a distribution. Representative p‐bit outputs for different *P*_STL_ of 25%, 50%, and 75% (right). (d) The shift of the sigmoid curve at various *V*_G_.

Before discussing the Potts model, the fundamental characteristics and the binary p‐bit operation of a single FB‐MOSFET are described. The left panel of Figure 1a shows a schematic diagram of the FB‐MOSFET and a photograph of a fabricated wafer. The device was fabricated on an 8‐inch silicon‐on‐insulator (SOI) wafer, ensuring large‐scale integration using CMOS technology. The FB is isolated by a buried oxide (BOX) layer, enabling the single‐transistor latch (STL) effect, which induces stochastic switching without an external random source. Under STL operation, impact ionization leads to hole accumulation in the FB, decreasing the potential barrier between the source and channel and, thus, causing abrupt switching from a high‐resistance state to a low‐resistance state [37]. This transition occurs at the latch voltage (*V*_latch_) of the *V*_D_, which varies stochastically due to impact ionization, resulting in the p‐bit behavior [26, 37]. It should be emphasized that, in an FB‐MOSFET with the same body doping concentration, the dominant mechanism responsible for generating holes stored in the FB under *V*_G_ near −2 V is impact ionization rather than band‐to‐band tunneling [38]. Furthermore, it has been confirmed that *V*_latch_ varies significantly with changes in the impact ionization rate [37]. Therefore, the observed stochastic switching originates from impact ionization rather than band‐to‐band tunneling. The right panel displays a schematic of the pulse train for *V*_D_, STL current, and a random p‐bit output through binarization. Figure 1b illustrates the experimental setup, comprising a top‐view scanning electron microscope (SEM) image of four FB‐MOSFETs sharing a common drain (left), a packaged chip image (middle), and a custom board featuring multi‐state p‐bit units (right). The results of binary p‐bit operations were also obtained in this experimental setup.

The drain current vs. drain voltage (*I*_D_–*V*_D_) characteristics at different *V*_G_s show that increasing *V*_G_ decreases the potential barrier, decreasing *V*_latch_ (Figure S1). Moreover, it confirms stochastic variations in *V*_latch_ and its dependence on *V*_G_ from the *I*_D_–*V*_D_ characteristics of ten consecutive cycles. Figure 1c shows the binary p‐bit operation, where *V*_D_ controls the STL probability (*P*_STL_). The left panel displays the sigmoidal fitting of *P*_STL_ across different *V*_D_ values (*V*_G_ = −2.0 V), while the right panel shows the time‐domain output voltages (*V*_out_) for three *V*_D_ settings (corresponding to *P*_STL_ values of 25%, 50%, and 75%). *V*_out_ is more likely to exceed the comparator threshold, producing the “1” state as *V*_D_ increases.

Figure 1d extends this analysis by examining *P*_STL_ at different *V*_G_ values. As *V*_G_ increases, *V*_latch_ decreases, shifting the sigmoid curve toward lower *V*_D_, adding an extra tunable variable compared to conventional two‐terminal p‐bit devices. Figure S2 shows sigmoid curves where the cycle‐to‐cycle variations at all *V*_G_s are represented as a distribution. Unlike two‐terminal devices, this structure allows independent tunability of multiple FB‐MOSFETs sharing a common drain, forming the building block for multi‐state p‐bit units. Table 1 provides a summary of the device‐level comparison between the proposed MOSFET‐based p‐bit and previously reported emerging device implementations, including MTJ‐, RRAM‐, and VO_2_‐based p‐bits [23, 26, 30, 31, 39, 40].

**TABLE 1:** Performance comparison of various p‐bits.

	This work	CTHP [30, 31]	SiO_x_ nano [39]	sMTJ [23]	LFSR [40]	Biristor [26]
Configuration	FB‐MOSFET	CuTe/HfOx/Pt	Au/SiOx/Pt	^a^complex	—	n‐p‐n
Number of levels	∼10	2	2	2	2	2
CMOS‐compatibility	full	limited	partial	limited	full	full
Power consumption	3.02 µW	^b^154 nW	4.06 µW	10 µW	^c^145 µW	^b^582 nW

^a^ Ta/Pt/[Co/Pt]7/Co/Ru/ [Co/Pt]2/Co/Ta/CoFeB/ MgO/CoFeB/Ta/Ru/Ta.

^b^ Power consumption was calculated, including the relaxation time of the p‐bits.

^c^ Calculated from the energy consumption, considering the clock frequency of 1 GHz.

### 2.2 Implementation and Operation of a Multi‐State p‐Bit Unit With the Potts Model

**FIGURE 2:**
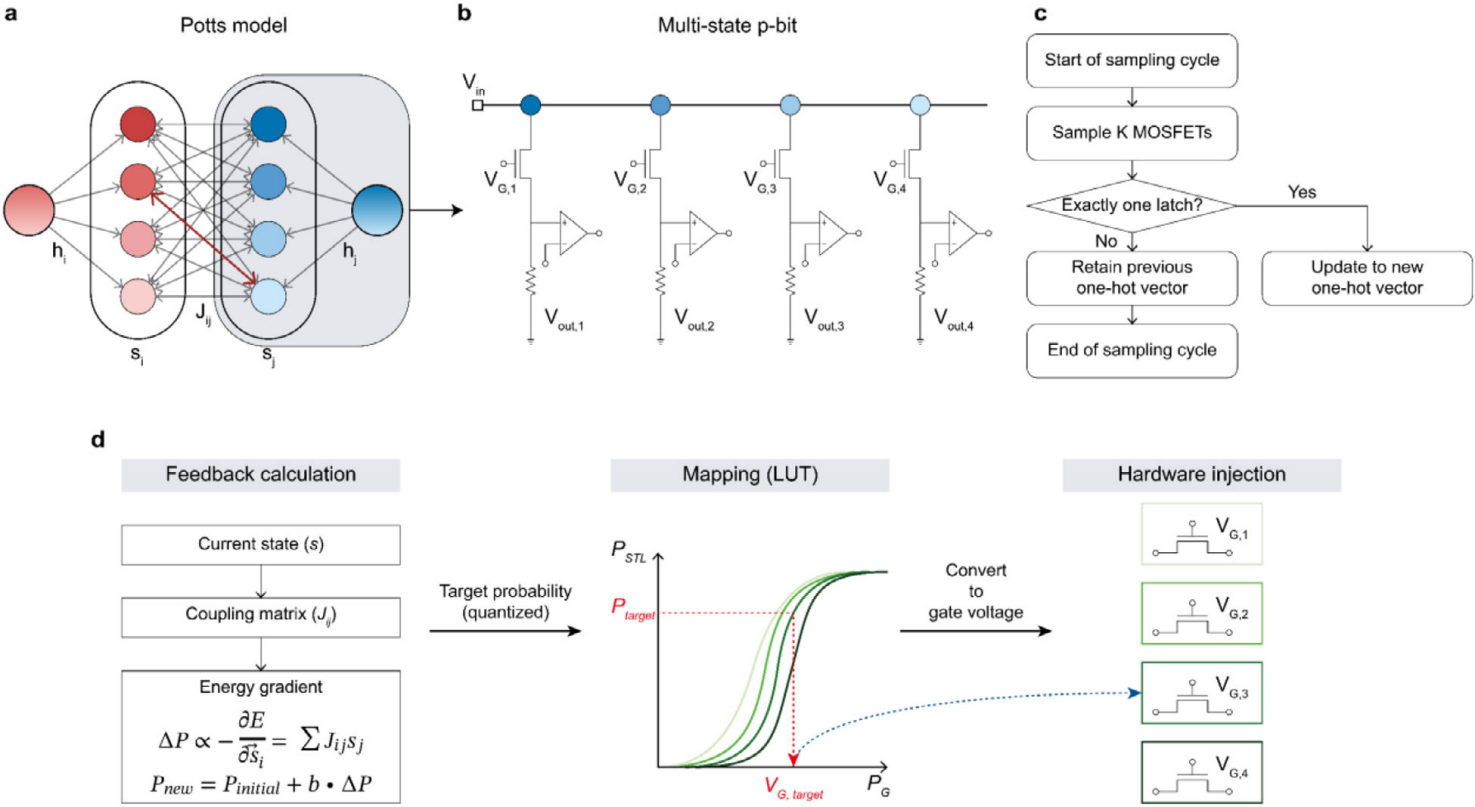
Multi‐state p‐bit operation with the Potts model. (a) Schematic diagram of the Potts model with four states in each site. (b) The circuit design for the multi‐state p‐bit unit. Each Potts node corresponds to a multi‐state p‐bit unit. (c) Flowchart of the one‐hot sampling method. Each cycle updates the node only when a latch occurs in a single device, while invalid cases keep the previous state. (d) Schematic of the feedback loop illustrating the feedback calculation and hardware injection processes through the gate voltage.

Figure 2 summarizes the implementation and operation of the multi‐state p‐bit unit for the Potts model. First, Figure 2a illustrates the Potts model, consisting of two 4‐state Potts nodes. Unlike the Ising model with *q* = 2 (Figure S3), the proposed Potts model uses *q* = 4. One‐hot states were sampled from the experimental setup, where multi‐state interactions and external bias define the Potts model. Each Potts node comprises four FB‐MOSFETs and four comparators, with a common drain terminal applying the input voltage (*V*_in_) to all FB‐MOSFETs, enabling multi‐state operation (Figure 2b). Although only four devices are described, the approach can be scaled to any number of *q* states by increasing the number of parallel‐connected FB‐MOSFETs. Moreover, while this work demonstrates proof‐of‐concept operation with a limited number of nodes, the same fabrication process can readily be expanded to wafer‐scale integration, as no process constraints limit the number of implementable p‐bit units. Figure S4 shows die‐to‐die uniformity of the fabricated 8‐inch wafer.

To govern the dynamic evolution of these states, a selective update protocol is implemented to ensure stable probabilistic operation. Figure 2c shows a flowchart of the one‐hot sampling method used in this work. In a single sampling cycle, the shared *V*_D_ triggers the parallel FB‐MOSFETs. The node state is updated to a new vector only when a one‐hot condition, in which exactly one device latches while others remain off, is detected. If multiple devices latch simultaneously or none latch, the system retains the previous valid one‐hot vector, ensuring stable probabilistic operation and preventing transient conflicts during updates. This mechanism naturally introduces asynchronicity into the update sequence, preventing large‐scale correlated flips.

Once the valid states are sampled, the system uses a feedback loop to translate the abstract Potts energy landscape into physical control parameters, as shown in Figure 2d. The feedback mechanism first computes the gradient of the energy function with respect to the spin state, derived from the interactions (Equation (2)). Based on this gradient, the update direction for the probability vector ($\Delta {/veP}_{{\vec{s}}_{i}}$) is set proportional to the negative gradient of the energy, ensuring the system evolves toward a lower‐energy state (Equation (3)).

(2) $$-\;\frac{\partial E}{\partial {\vec{s}}_{i}}=-\sum_{j}^{N}A_{i,j}{\vec{s}}_{j}$$

(3) $$\Delta {\vec{P}}_{{\vec{s}}_{i}}\propto -\sum_{j}^{N}A_{i,j}{\vec{s}}_{j}$$

The probability vector is then scaled by a time‐dependent annealing parameter *b* and accumulated onto the base probability to define the target probability for the subsequent iteration (Equation (4)).

(4) $${\vec{P}}_{{\vec{s}}_{i}}={\vec{P}}_{initial}+b\Delta {\vec{P}}_{{\vec{s}}_{i}}$$

Finally, this target probability is converted into the specific *V*_G_ required for each FB‐MOSFET using a pre‐calibrated look‐up table (LUT). This mapping relies on the experimentally measured sigmoidal *P*_STL_‐*V*_G_ characteristic of the device, ensuring that the mathematical update dynamics derived from the Potts model are accurately realized in the multi‐state p‐bit hardware.

In the proposed system, the Potts spin state (*s*) correspond to the output state of each multi‐state p‐bit. The energy contribution, determined by the coupling (*J*) and bias (*h*) terms, is calculated and then physically applied to the device as *V*_G_, which controls the probabilistic switching behavior of the p‐bit. The drain terminal voltage (*V*_in_) is not directly related to any specific parameter in the Potts model but can act as a global control for annealing or temperature scaling to adjust the overall system energy. In this work, *V*_in_ was kept constant to verify the inherent stochastic behavior of the FB‐MOSFET‐based multi‐state p‐bits, while future implementations may dynamically vary *V*_in_ to manage the annealing process. In the multi‐state p‐bit circuit, the four *V*_G_s (*V*_G,1_ to *V*_G,4_) are not adjusted independently but are collectively determined through weighted feedback from neighboring nodes via the Potts coupling matrix (*J*). Each node's probabilistic state results from the combined influence of these *V*_G_s, rather than individual control.

**FIGURE 3:**
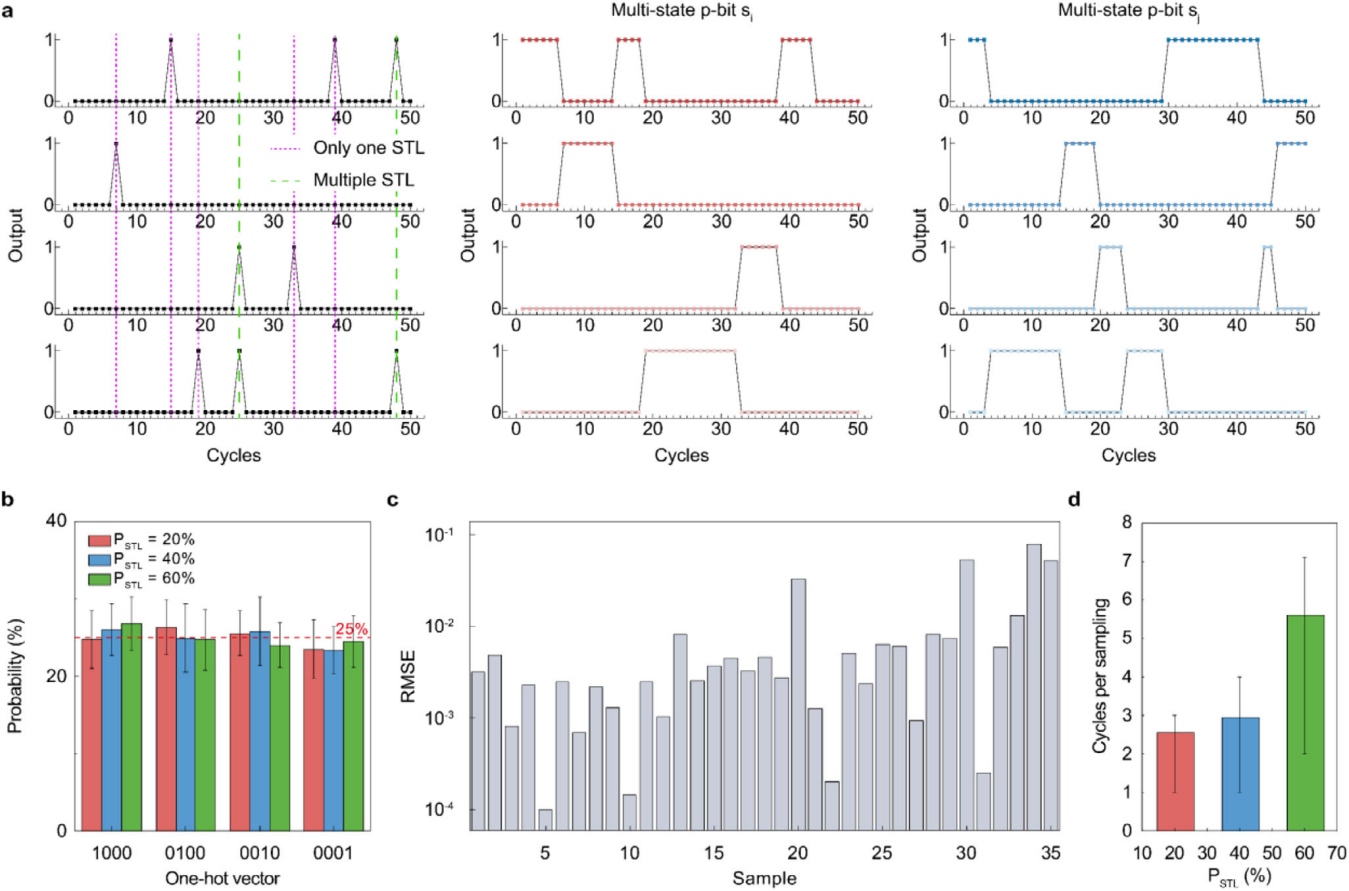
Experimental verification of the stochastic one‐hot sampling. (a) The one‐hot sampling process in a 4‐state p‐bit unit. Each FB‐MOSFET operates under 5% STL conditions (left). Before the first one‐hot sampling occurs, all Potts nodes are set to state [1000]. When one‐hot sampling occurs (purple dashed line), the state of the Potts node is changed to a corresponding one‐hot vector (middle). When multiple devices show latch at the same time (green dashed line), the Potts node retains its current state. In the same way, Potts node *s*_j_ outputs one‐hot sampling results (right). (b) Output probability of each one‐hot vector when sampling was performed 100 times for 10 epochs. For each *P*_STL_, all one‐hot vectors showed similar probabilities. (c) RMSE for all sample combinations, where the *P*_STL_ of four FB‐MOSFETs is quantized by 20%. (d) Cycles per sampling as a function of *P*_STL_. As *P*_STL_ increases, sampling a one‐hot sample requires more cycles because of a higher collision likelihood.

Figure 3a describes the one‐hot sampling process. The left panel shows the binary outputs (“0” or “1”) of each device over time for Potts node *s*_i_ with *V*_in_ = 3 V and *V*_G_ = −2 V for all FB‐MOSFETs (*P*_STL_ = 5%), and 50 *V*_in_ pulses applied. Before the one‐hot sampling operation, both nodes, *s*_i_ and *s*_j_, are set to state [1000] to initialize the system, and they remain in the [1000] state up to the sixth and third cycles, respectively, in the middle (*s*_i_ node) and right (*s*_j_ node) panels. In the seventh cycle, for example, only device #2 in node *s*_i_ generates a “1” state, while the rest generate a “0” state. Therefore, sampling is performed in the seventh cycle, where the one‐hot vector output becomes [0100]. The middle panel shows one‐hot sampling results in node *s*_i_ based on the suggested method. The [0100] is retained up to the 15th cycle when device #1 generates a “1” state while all others generate a “0” state. Then, the one‐hot vector output becomes [1000] (represented by the purple dashed line). It should be noted that devices #3 and #4 generate a “1” state simultaneously at the 25th cycle, so the output does not change to [0100] or [0001] (as indicated by the green dashed line). Similarly, the right panel of Figure 3a displays the one‐hot sampling result for the node *s*_j_.

To validate the stochastic performance and tunability of the multi‐state p‐bit unit, a comprehensive statistical analysis was conducted. First, Figure 3b demonstrates the system's ability to maintain a uniform probability distribution. Even when the base *P*_STL_ varies from 20% to 60%, the output probability for each one‐hot vector consistently aligns with the ideal 25%. Moreover, the precise tunability of the multi‐state p‐bit unit was verified by analyzing cases where different *V*_G_ values are applied to each FB‐MOSFET to generate non‐uniform distributions, as shown in Figure 3c. To assess this performance, all 35 possible combinations, derived from a 4‐state node, where each device is assigned a quantized probability level (0.2, 0.4, 0.6, or 0.8), were tested. By calculating the root‐mean‐square error (RMSE) between the theoretically derived ideal probabilities and the experimentally measured results for these combinations (Note S1), it was confirmed that the system can accurately map the target probability with minimal error. Finally, Figure 3d shows the sampling latency as a function of *P*_STL_. As *P*_STL_ increases, the probability of multiple devices latching simultaneously (collision) rises, which increases the average number of cycles required to capture a valid one‐hot sample. These results indicate that the update intervals may differ across nodes.

In this system, the node was updated only when a one‐hot sample was obtained in a single cycle; otherwise, it maintained the previous state. As it randomly provides asynchronous updates to each node, only a limited subset of nodes are updated in each iteration, yielding a randomized, scalable annealing schedule. Therefore, it provides a parallel update on a synchronous clock, using annealing to solve COPs. It should be noted that the term “synchronous clock” refers only to the presence of a global timing reference used for measurement, not to synchronous spin‐update scheduling. Node updates occur asynchronously because only nodes that yield valid one‐hot samples in a given cycle are updated; others maintain their previous state. Therefore, even with a global clock, the update sequence across nodes remains randomized and inherently asynchronous, ensuring natural annealing behavior and preventing large‐scale correlated flips. Many previous studies have used various methods to achieve asynchronicity [19, 24, 25, 35].

The proposed method updates a node only when a one‐hot sample is obtained in a single cycle; otherwise, it maintains the previous state. This approach yields a higher success probability and lower latency than methods that wait for all nodes to update. Its effectiveness for complex COPs is further discussed in Section 2.3 and in the related Figure S1. Also, Figure S5 and Note S2 illustrate another advantage of the Potts model over the Ising model for graph duplication in complex COPs.

### 2.3 Experimental Results of Spin‐Glass and Max‐K‐Cut Problems

**FIGURE 4:**
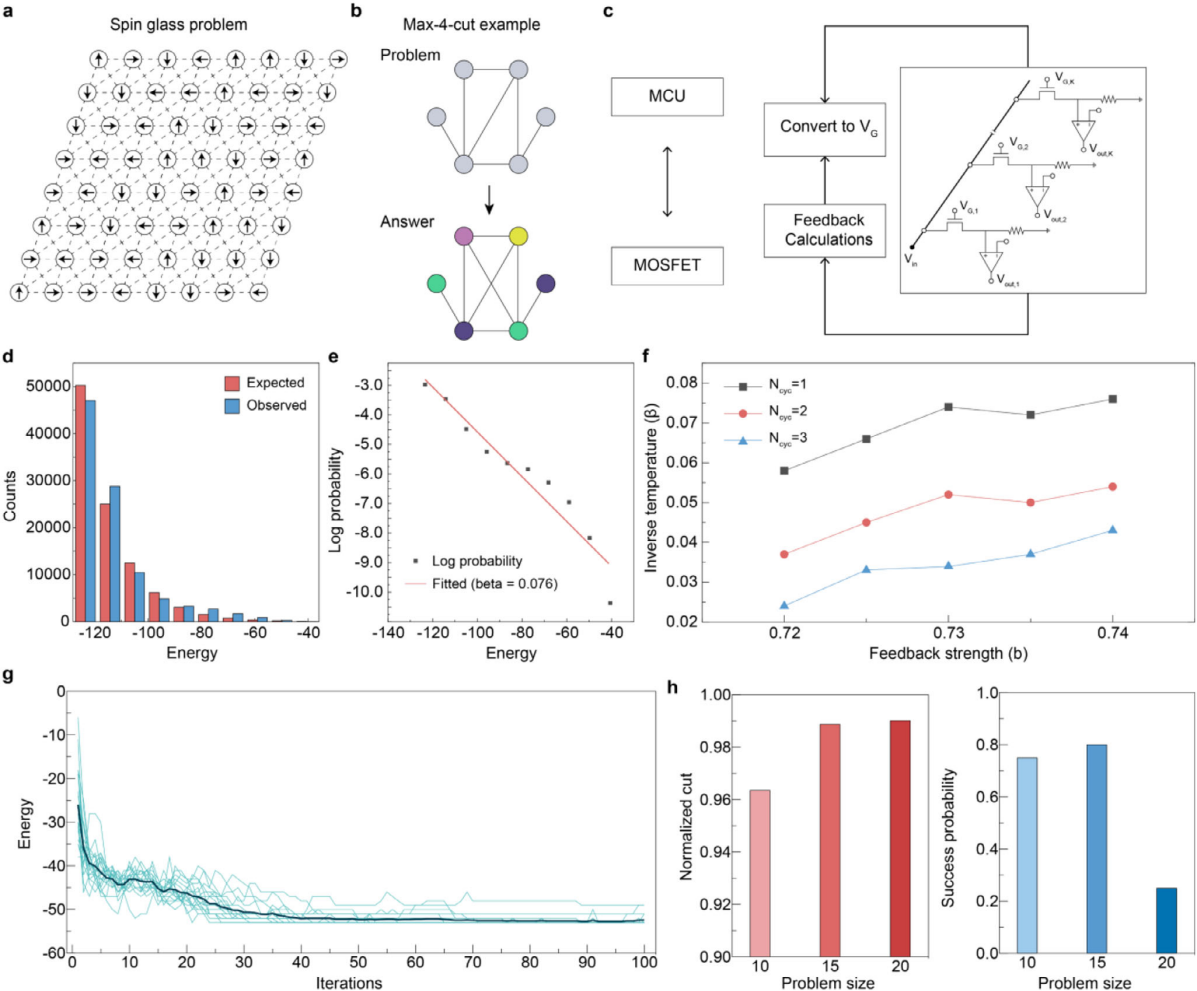
Experimental results of spin‐glass and max‐K‐cut problems. (a) An example of a spin glass problem on an 8 × 8 grid. (b) An example of a max‐4‐cut problem and its answer. (c) Experimental setup for a max‐K‐cut problem. After the one‐hot sampling from the circuit on the right, *V*_G_ for feedback was calculated to control the subsequent iteration. (d) Comparison between the energy observed in 2000 trials (blue) and the theoretical energy distribution (red). (e) Linear fit of energy vs. the logarithm of its distribution derived from (d). (f) Inverse temperature (*β*) from different *N*_cyc_s and feedback strength (*b*). Larger *N*_cyc_ leads to higher temperatures, whereas increased *b* leads to lower temperatures. (g) Energy profiles of solving a 15‐node max‐4‐cut problem. The average energy from 20 independent trials is shown. (h) Normalized cut value and success probability for solving max‐4‐cut problems with different problem sizes. Each parameter was derived from 20 measurements.

Spin glass and max‐4‐cut problems were solved experimentally to evaluate the suggested method [13, 20]. The two problems are similar in that they are described as Potts nodes with coupling matrices (Figure 4a,b). First, the spin glass problem involves disordered spin interactions, in which the spin states correspond to Potts nodes. In this problem, nodes are arranged in an 8 × 8 grid, and interactions exist only between spatially adjacent nodes. Second, in the max‐4‐cut problem, each node is assigned one of the four colors to maximize the number of cuts between nodes of different colors. Both problems share the same energy function described by Equation (5), where the adjacency matrix, *A*_*i*, *j*_, defines node‐to‐node connections within the problem graph. These two graph problems were transformed into energy‐based problems using Equation (1), with coupling encoded as an adjacency matrix and no bias. In this work, unlike previous studies with the binary spin states, each spin has 4 states.

(5) $$E=-\sum_{i<j}^{N}A_{i,\;j}\left(1-{\vec{s}}_{i}\cdot {\vec{s}}_{j}\right)$$

Note S3 describes how COPs, such as max‐cut and spin‐glass problems, are solved using the Ising model with annealing. It offers background for understanding the Potts model approach discussed below.

Each Potts node is composed of four drain‐shared FB‐MOSFETs, which determine its state based on a one‐hot vector output ([1000], [0100], [0010], [0001]). Figure 4c illustrates the experimental setup, featuring a custom board and a microcontroller unit (MCU) for both problems. Figure S6 and Note S4 provide further details of the custom board and sampling process, respectively. Also, additional details on the coupling and update mechanism of multi‐state p‐bits, particularly focusing on how spin states are updated in parallel using a LUT‐based architecture, are shown in Figure S6.

First, a spin‐glass problem on an 8 × 8 grid was solved to validate the Boltzmann distribution and to estimate the appropriate annealing temperature for the proposed multi‐state p‐bit system. In the system described by Equation (5), the *P*_STL_ values of FB‐MOSFETs in each Potts node were calculated as a function of *V*_G_, which was quantized to 10 levels to minimize the hardware overhead (Experimental Section). The results were then fed back to the nodes. Specifically, the contribution of each p‐bit to the energy was derived by integrating interactions with neighboring nodes (Note S5). Figure 4d shows that, over 2000 trials, each with 50 iterations, the system sampled random states in which the energy calculated from Equation (5) satisfies the Boltzmann distribution. Figure 4e further validates this finding by plotting the logarithm of the observed probability (log P(E)) against energy (E), which demonstrates a linear relationship. The red line represents the theoretical Boltzmann fit, with an estimated inverse temperature *β* = 0.076.

Moreover, this inverse temperature was controlled through various parameters corresponding to the annealing process. The increase in parameter *b*, which is the coefficient of the feedback signal (Note S5), resulted in an effective decrease in temperature due to the system's faster saturation. Meanwhile, the other parameter, *N*_cyc_, refers to the number of cycles waited for one‐hot sampling. A single cycle described in Section 2.2 exhibited the highest *β* (the lowest temperature), as most nodes remained in their previous states. However, longer cycles led to lower *β* (higher temperature), as more nodes were allowed to flip in additional cycles. Consequently, the suggested method could sample from the Boltzmann distribution with controllable temperature (Figure 4f).

Then, max‐4‐cut problems were solved to evaluate the annealing method for solving COPs. Similar to the spin glass problem, *V*_G_ of each FB‐MOSFET in the corresponding p‐bit was tuned according to the energy in Equation (5). In addition to the process from Figure 4d–f, the feedback parameter *b* was increased linearly throughout the iterations to gradually lower the temperature and guide the system toward the ground state (Note S5) [41, 42]. Figure 4g shows the average energy histories obtained from 20 independent max‐4‐cut experiments on a 15‐node graph using the setup in Figure 4c. Here, four 4‐state p‐bit (total 16 FB‐MOSFET) were used to perform the experiments using the sequential sampling process. The system consistently converges to the ground state, demonstrating its effectiveness in handling complex COPs. Figure 4h shows the normalized cut values and success probabilities for various problem sizes, demonstrating that the system is applicable for larger problem sizes.

**FIGURE 5:**
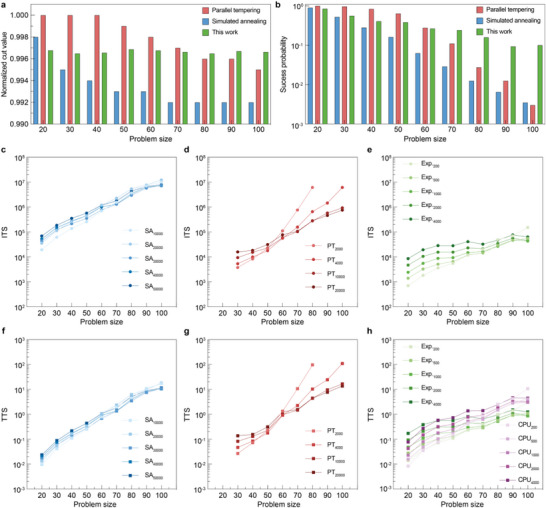
Simulation results of max‐4‐cut problems. (a,b) Normalized cut value and success probability of three algorithms on max‐4‐cut problems, simulated annealing (SA), parallel tempering (PT), and this work. For each algorithm, iterations of 10 000, 4000, and 1000 were chosen, considering the execution times. Each problem size consists of 20 instances, each solved 100 times. (c–e) Iteration‐to‐solution (ITS) of the three algorithms with various iterations. The ITS values omitted in the figure correspond to scenarios in which the success probability is 100% for a problem size of 20% or 0% for larger problem sizes. The lowercase number indicates the number of iterations for each dataset. (f–h) Time‐to‐solution (TTS) of the three algorithms with various iterations. The TTS values were calculated by multiplying the execution time by ITS in (c–e).

In this process, device‐to‐device variations between devices can have a negative impact, as different devices may exhibit different probability distributions. Figure S7 and Note S6 show that *V*_G_ modulation can mitigate such variations by shifting the sigmoidal curves by an amount corresponding to the device variation offset. Furthermore, Figures S8 and S9 show the experimental results for solving max‐K‐cut problems (*K* = 3–10) to verify the feasibility of the generalized multi‐state p‐bit unit. For example, 10 FB‐MOSFETs were used to solve the max‐10‐cut problem via sequential sampling. The results show that the suggested one‐hot sampling method enables synchronous updates in Potts models with more states (*K*). In summary, Figure 4 shows the experimental results from physical measurements on the fabricated FB‐MOSFET array and the custom PCB operated at 50 kHz. Although stable hardware demonstration results exceeding 50 kHz were not obtained for the full system, the 4‐state p‐bit operation at 1 MHz was measured, demonstrating that the speed limitation does not originate from the devices but from circuit parasitics (Figure S10). In contrast, Figure 5 displays simulation‐based analyses that use experimentally extracted device parameters to assess the system‐level performance of the proposed multi‐state p‐bit architecture.

### 2.4 Simulation Results and Comparison With Conventional Optimization Algorithms

To further evaluate the suggested multi‐state probabilistic computing and compare it to conventional optimization algorithms, max‐4‐cut problems with larger sizes were simulated (Figure 5). In addition to the suggested multi‐state p‐bit network, simulated annealing and parallel tempering were evaluated for comparison. The conventional codes for the two algorithms were modified for the Potts model (Experimental Section). Figure 5a,b show normalized cut values and success probabilities for various problem sizes. Although the suggested method exhibits similar performance in small problem sizes (up to ∼60), it outperforms the others in larger problem sizes due to scalability in annealing via one‐hot sampling (Figures S11 and S12). Figure 5c–e show the iteration‐to‐solution (ITS) required to guarantee a 99% success probability for algorithms with varying iteration counts [42]. The two conventional algorithms require at least thousands of iterations to solve large problems, whereas the proposed method converges in ∼200 iterations, decreasing the ITS by two orders of magnitude.

Then, time‐to‐solution (TTS) was evaluated for each method, and Figure 5f–h show the results. The suggested method yielded the lowest value among the algorithms executed on the central processing unit (CPU). Moreover, it exhibited a TTS similar to that of the multi‐state p‐bit algorithm executed on the CPU, assuming a clock frequency of 50 kHz in the experimental setup. It also showed a better scaling trend, suggesting that the multi‐state p‐bit offers scalable solutions in dedicated hardware and CPU. All results were obtained from C code executed on an Intel Xeon Silver 4210R CPU (see Experimental Section). Furthermore, to evaluate scalability, a max‐4‐cut problem with 60 nodes was solved, showing performance comparable to conventional CPU algorithms in terms of execution time. Detailed discussions of potential speedups from full on‐chip integration are provided in Figure S13 and Note S7. This projection is based on the nanosecond‐scale stochastic switching speed of FB‐MOSFETs previously reported [43].

Lastly, Table 2 presents a comparison of this work with various Ising machines, including the Hopfield network [41, 44] and oscillatory neural networks [33, 34, 45]. The table shows TTS and energy‐to‐solution (ETS) performance metrics in solving unweighted max‐cut problems with 100 nodes and a density of 0.5 (Note S8). The performance metrics were obtained by assuming a max‐4‐cut with 50 nodes for the proposed method, accounting for the problem‐space difference. This consideration is because the N‐node max‐4‐cut problem has the same problem space as a 2N‐node max‐cut problem (Figure S14). Furthermore, Figure S15 shows the effect of various *N*_cyc_. The proposed method demonstrates superior predicted performance in the two metrics, and many states can be implemented for various COPs that other approaches cannot solve. Moreover, its application can be extended to other probabilistic computing paradigms, such as invertible logic and integer factorization. Table S1 provides a detailed calculation of energy consumption. Furthermore, compared to other devices used in previous works, this work utilizes fully CMOS‐based p‐bits that resemble the NOR flash array in structure, which has already been commercialized with a capacity of up to 1 Gb [46, 47]. Therefore, the proposed method is expected to expand device density readily.

**TABLE 2:** Performance comparison of the present Potts machine with various Ising machines.

	This work	CPU (parallel tempering)	Mem‐HNN [41]	In‐material [44]	ONN (ring oscillator) [33]	ONN (PTNO) [34]	ONN (VO_2_) [45]
Connectivity	All‐to‐all	All‐to‐all	All‐to‐all	All‐to‐all	All‐to‐all	All‐to‐all^a^	All‐to‐all^a^
Number of states	∼10	∼10	2	2	2	2	2
CMOS‐based	o	o	⨯	⨯	⨯	⨯	⨯
Solved problem	Max‐4‐cut	Max‐4‐cut	Max‐cut	Max‐cut	Max‐cut	Max‐cut	Max‐cut
TTS^b^	5.6 µs (50 nodes)	9 ms (parallel)	25 µs	26 µs	>23 µs	30 µs	—
TTS^c^	875 µJ	45 mJ	400 µJ	895 µJ	924 µJ	768 µJ	—

^a^ Coupling of oscillators in electrical impedance shows limited reconfigurability.

^b^ TTS was calculated for solving unweighted max‐cut problems with 100 nodes and a density of 0.5.

^c^ ETS was obtained from a specific hardware implementation of each work, where some studies assumed simpler hardware than was required to solve problems with 100 nodes.

## 3 Conclusions

Recent research in probabilistic computing has made significant progress, with most efforts focused on binary p‐bit operations. However, studies on the Potts model remain in their early stages despite the density of p‐bits being crucial for practical large‐scale problems. Previously studied two‐terminal devices, such as MTJs or RRAMs, cannot precisely manipulate the device's state, especially for a multi‐state p‐bit. Moreover, the fabrication processes for these devices are relatively immature compared to those for MOSFETs. This work demonstrated multi‐state p‐bit systems that can be fully integrated with CMOS technology, significantly expanding the capabilities of probabilistic computing. The tunable multi‐state p‐bit unit, utilizing the stochastic behavior of FB‐MOSFETs, can efficiently solve complex COPs using common drain sharing and a one‐hot sampling method. The intrinsic asynchronicity of the one‐hot sampling method enabled scalable annealing schedules and efficient parallel updates with minimized simultaneous node flips, thereby mitigating convergence bottlenecks.

The effectiveness of this approach was validated by experimentally solving spin glass and max‐4‐cut problems using a custom board with an MCU. Furthermore, the superiority of the proposed method was established through a comprehensive comparison of simulation results with those of different optimization algorithms using the ITS and TTS metrics across various problem sizes. The potential of this work as a next‐generation probabilistic computing platform is validated through comparisons with CPU and various previous studies from the perspectives of TTS and ETS. This progress indicates the potential for integrating multi‐state p‐bits into large‐scale CMOS‐compatible architectures, offering an energy‐efficient solution for various computationally hard problems.

## 4 Experimental Section

### 4.1 Device fabrication

The FB‐MOSFETs were fabricated on an 8‐inch p‐type (100) SOI wafer (SOITEC, G8P‐297‐01) with a BOX layer of 145 nm and a top silicon layer of 55 nm. The channel regions were defined through photolithography and plasma etching. Subsequently, the gate dielectric and poly‐crystalline silicon gates were deposited and patterned using photolithography and plasma etching. Afterward, source and drain implantation and rapid thermal annealing were performed. Finally, an interlayer dielectric and aluminum were deposited for metallization. The aluminum was patterned using a lift‐off process to interconnect the drains of multiple FB‐MOSFETs, completing the multi‐state p‐bit unit.

### 4.2 Electrical Characterization

The output characteristics of the FB‐MOSFETs were measured using a semiconductor parameter analyzer (HP4145B, Hewlett‐Packard), controlled through a LabVIEW‐developed interface. A custom board was built with a digital‐to‐analog converter (DAC) and an analog‐to‐digital converter (ADC) for the pulse measurements. The multi‐state p‐bit circuit, comprising drain‐shared FB‐MOSFETs and comparators, was also implemented on a custom board.

### 4.3 Measurement Setup

The test system for the experimental demonstration consisted of a host PC, a custom analog and digital mixed circuit mounted on a printed circuit board (PCB), and an external 4‐channel oscilloscope (OSC). The host PC managed the measurement interface, controlled the MCU connected to the board, and processed the measurement results received via serial communication. An ARM Cortex‐based MCU processed control commands from the host PC and subsequently transmitted them to the board's integrated circuit (IC) components according to their intended purposes. The board included regulators for stable positive and negative power supplies to IC components and switch matrices for selecting devices and their corresponding terminals. Two 4‐channel DACs were used to bias independent voltages to the gate and drain of the device, respectively, and to generate adjustable pulses. Current outputs from the source electrode were converted into voltages and then delivered to the MCU's internal 12‐bit ADC or an external oscilloscope (OSC). Various amplifiers on the board were connected with adjustable passive resistors and utilized as transimpedance and inverting amplifiers. The devices wire‐bonded in a ceramic dual‐in‐line package substrate were directly connected to the device‐under‐test socket on the board. The host PC interface, the control code, and the embedded code for the MCU were all written in Python.

### 4.4 Simulation of Various Optimization Algorithms

The simulation of COPs using multi‐state p‐bit units, simulated annealing, and parallel tempering was carried out based on the equations provided in Note S5 and implemented in Python and C. First, the multi‐state p‐bit unit based on drain‐shared FB‐MOSFETs was modeled using measurements, incorporating variations. One‐hot sampling of the measurement data was used for derivative calculations and proxy updates. These updates are converted to *V*_G_ from the measured data. *V*_G_ was quantized to 10 probability levels to minimize overhead in a hardware implementation using a look‐up table. Finally, the modulation schedule for the linear parameter (*b*) was tuned for each problem size.

Both simulated annealing and parallel tempering codes were based on the conventional algorithm, but with different energy calculations. First, simulated annealing was implemented by lowering the initial temperature (*T*_0_) by multiplying a constant (*α*). The two parameters were fine‐tuned for problem size and the number of iterations used to solve max‐4‐cut problems. For the parallel tempering, 20 replicas were assumed, and the initial temperatures were fine‐tuned similarly. All algorithms were optimized for the problem set shown in Figure 4. For each problem size, 20 random instances with density 0.5 were generated in the problem set.

### 4.5 Performance Evaluation of Various Optimization Algorithms

Three optimization algorithms were implemented in C to evaluate execution time. A Linux server with an Intel Xeon Silver 4210R CPU was used for the evaluation. 10–100 trials are executed in parallel for each algorithm to minimize variation across trials. Then, the measured time was divided by the number of parallel runs to obtain the final values. For the evaluation in Figure 5, the first instance, which consisted of 60 nodes from the problem set in Figure 4, was used.

## Conflicts of Interest

The authors declare no conflicts of interest.

## Supporting information




**Supporting File**: adma72593‐sup‐0001‐SuppMat.docx.

## Data Availability

The data that support the findings of this study are available from the corresponding author upon reasonable request.
