# Supplementary material for: Multi‐State Probabilistic Computing Using Floating‐Body MOSFETs Based on the Potts Model for Solving Complex Combinatorial Optimization Problems
Source: Adv Mater. 2026 Feb 17;38(16):e16797. doi: 10.1002/adma.202516797 (PMC12994325; doi:10.1002/adma.202516797)
Supplement: Supplementary file 1 — Supporting File: adma72593‐sup‐0001‐SuppMat.docx. [file ADMA-38-e16797-s001.docx]

SUPPORTING INFORMATION

Multi-state Probabilistic Computing using Floating-body MOSFETs based on the Potts model for Solving Complex Combinatorial Optimization Problems

*Sunwoo Cheong^1,†^, Soo Hyung Lee^1,†^, Janguk Han^1,†^, Jun-Young Park^1^, Dong Hoon Shin^1^, Yoon Ho Jang^1^, Sung Keun Shim^1^, Sungho Kim^1^, Cheol Seong Hwang^1,^*, and Joon-Kyu Han^1,^**

^1^Department of Materials Science and Engineering and Inter-university Semiconductor Research Center, College of Engineering, Seoul National University, Seoul, 08826, Republic of Korea

^†^ These authors contributed equally to this work.

* Corresponding author (e-mail: *cheolsh@snu.ac.kr, joonkyuhan@snu.ac.kr)*

Table of contents

- Supporting Figures S1 ~ S15
- Supporting Table S1
- Supporting Notes 1 ~ 8
- Supporting References


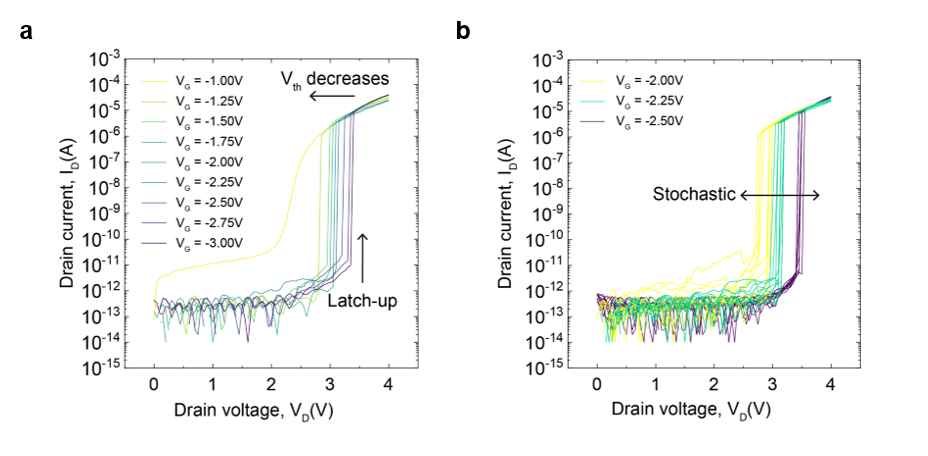


Figure S1 | P-bit operation of FB-MOSFET.

(a) The shift of latch-up voltage (*V*_latch_) at various gate voltages (*V*_G_). *V*­_latch_ decreases as *V*_G_ increases because the initial potential barrier between the source and FB decreases. (b) *V*_latch_ fluctuations at various *V*_G_ from 10 consecutive cycles caused by irregular impact ionization.


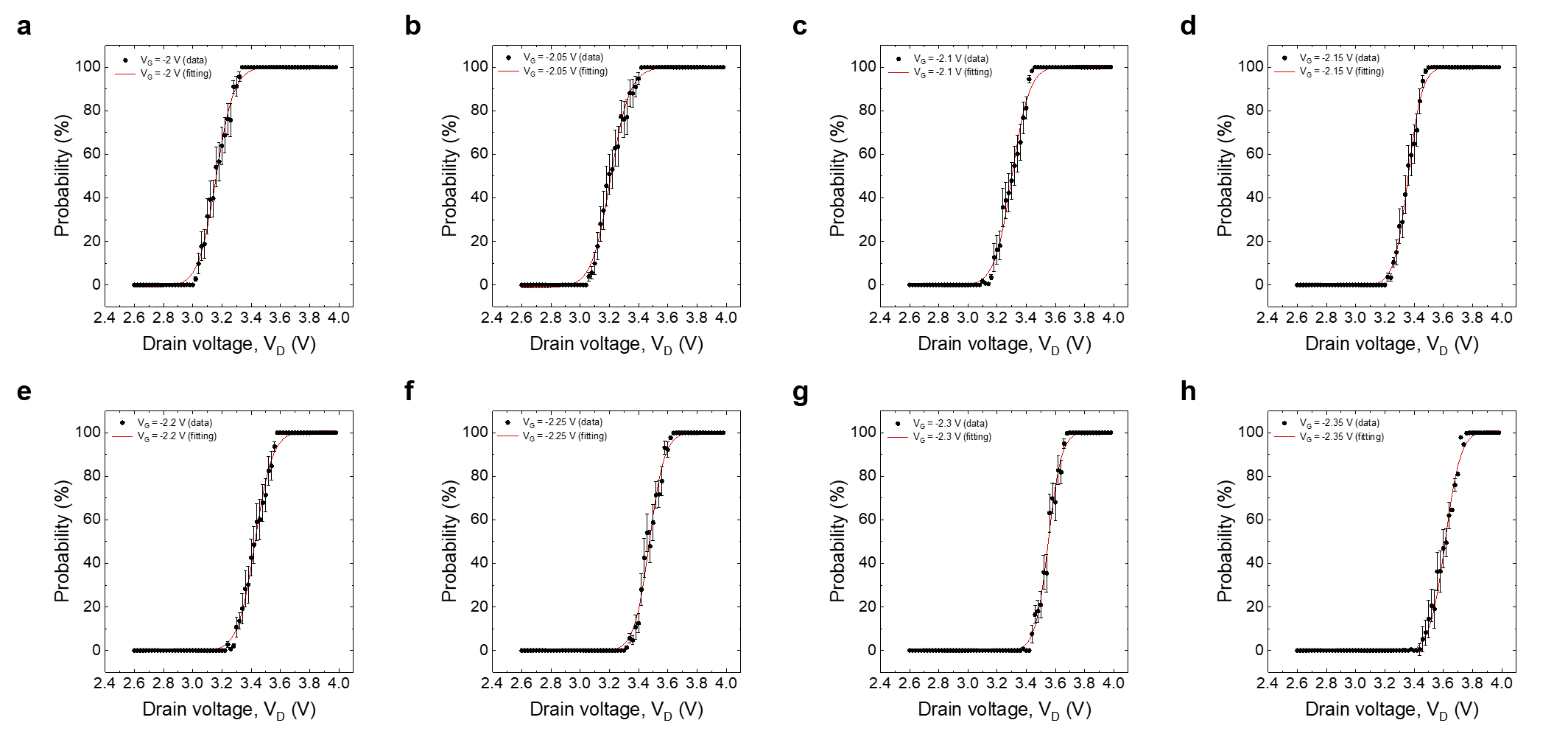


Figure S2 | Cycle-to-cycle variation at various gate voltages (*V*_G_).

The probability of STL versus drain voltage (*V*_D_) showing cycle-to-cycle variation at *V*_G_ of (a) -2.00 V, (b) -2.05 V, (c) -2.10 V, (d) -2.15 V, (e) -2.20 V, (f) -2.25 V, (g) -2.30 V, and (h) -2.35 V.


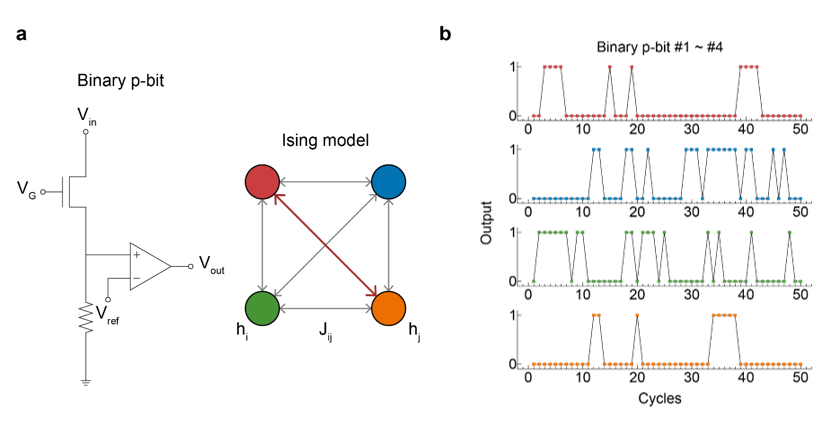


Figure S3 | Binary p-bit operation of FB-MOSFET with the Ising model.

(a) The circuit design for the binary p-bit unit and schematic diagram of the Ising model with 4 nodes. Each node corresponds to a binary p-bit unit. (b) Sampling results for four binary p-bits.

Figure S4 | Die-to-die uniformity in 8-inch wafer.

Histogram showing the average *V*_latch_ distribution of 128 randomly selected dies out of 256 total dies on an 8-inch wafer. For each selected die, *V*_latch_ was measured from five FB-MOSFET devices and averaged.


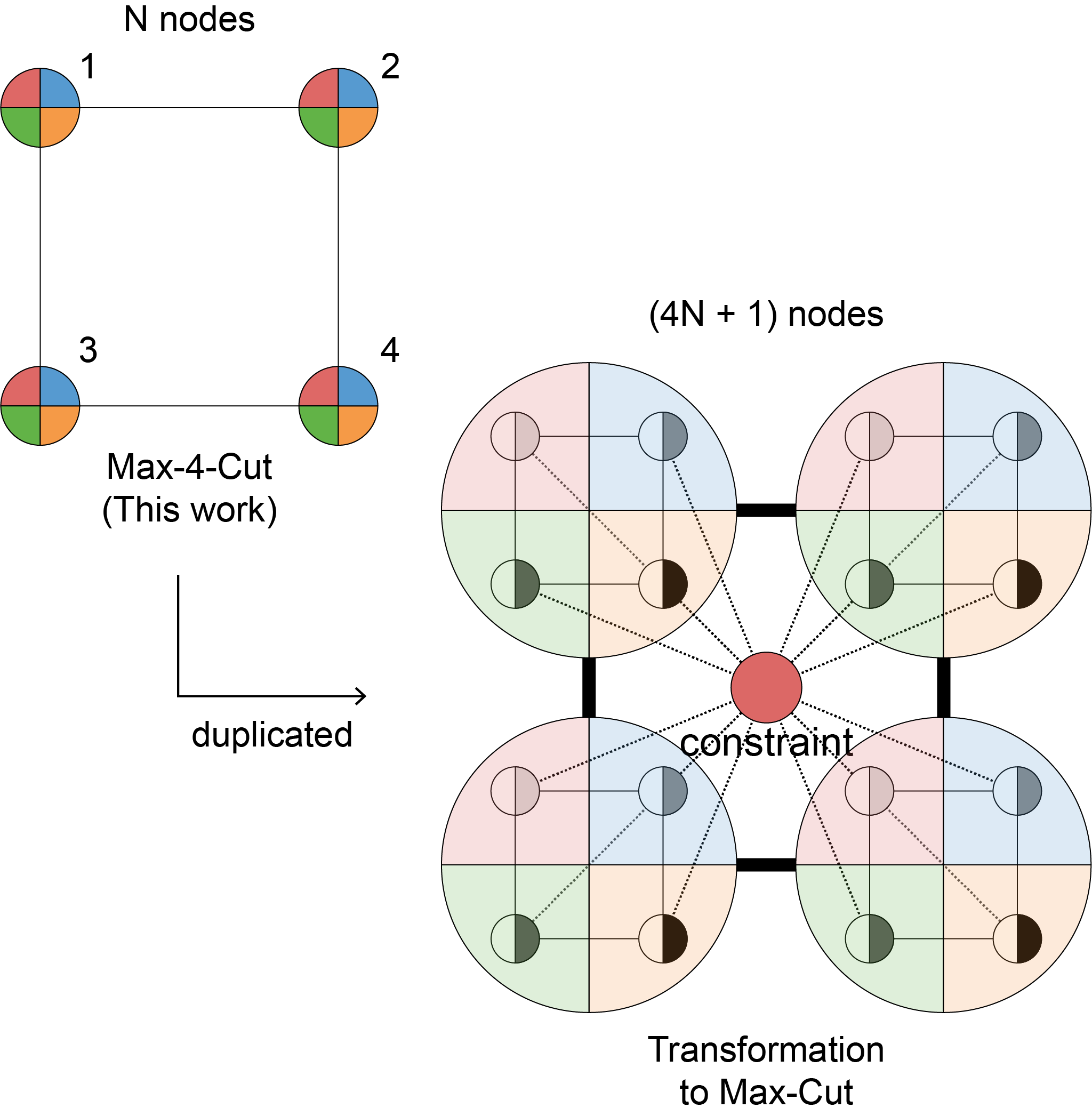


Figure S5 | Graph duplication from max-4-cut to max-cut.

A transformation of a 4-node max-4-cut problem into a 17-node max-cut problem. Duplicated nodes with the additional constraint are shown. A detailed explanation of this problem is provided in Supporting Note 2.


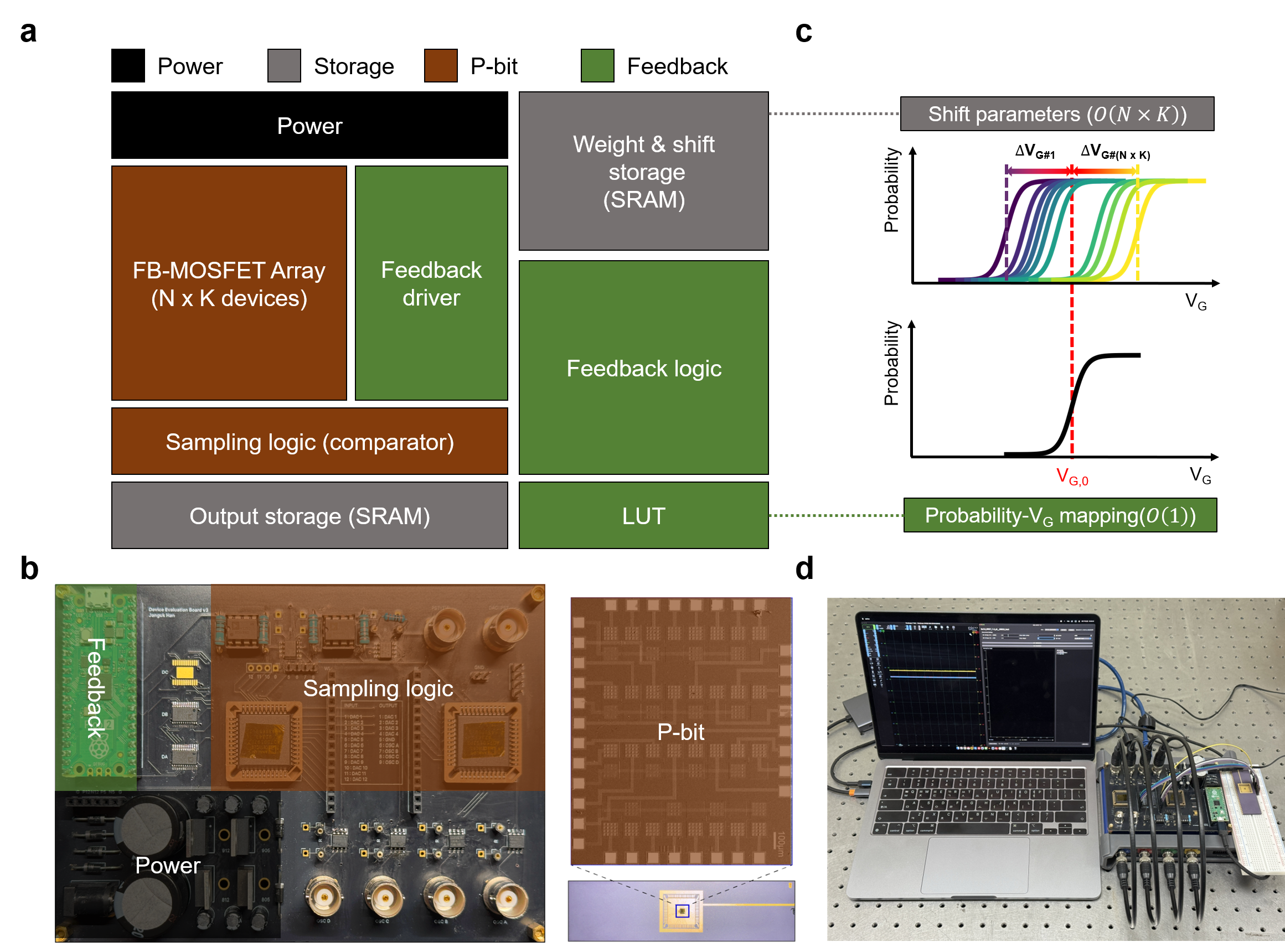


Figure S6 | Circuit diagram and experimental setup for multi-state p-bit demonstration.

(a) Circuit diagram of the suggested method. The schematic is composed of four parts for power supply, storage, p-bit array, and feedback logic. (b) Corresponding PCB implementation of the schematic in (a). Output, weight and shift parameter storage are stored in the host CPU. (c) Shift parameters and probability-to-*V*_G_ mapping. For *N* nodes with *K* levels, *N* x *K* nodes exhibit shifted sigmoidal curves in Probability-*V*_G_ plots. The shifted amounts are saved as shift parameters ($\Delta$*V*_G_) for each FB-MOSFET, resulting in O(*N* x *K*) shift parameters stored in SRAM. After the calibration (lower panel), probability-to-*V*_G_ mapping of all the devices can be saved to align sigmoidal curves to center around *V*_G,0_. However, duplicated LUTs should be adopted for fully parallel calculation. (d) Photographs of the experimental setup and user interface.


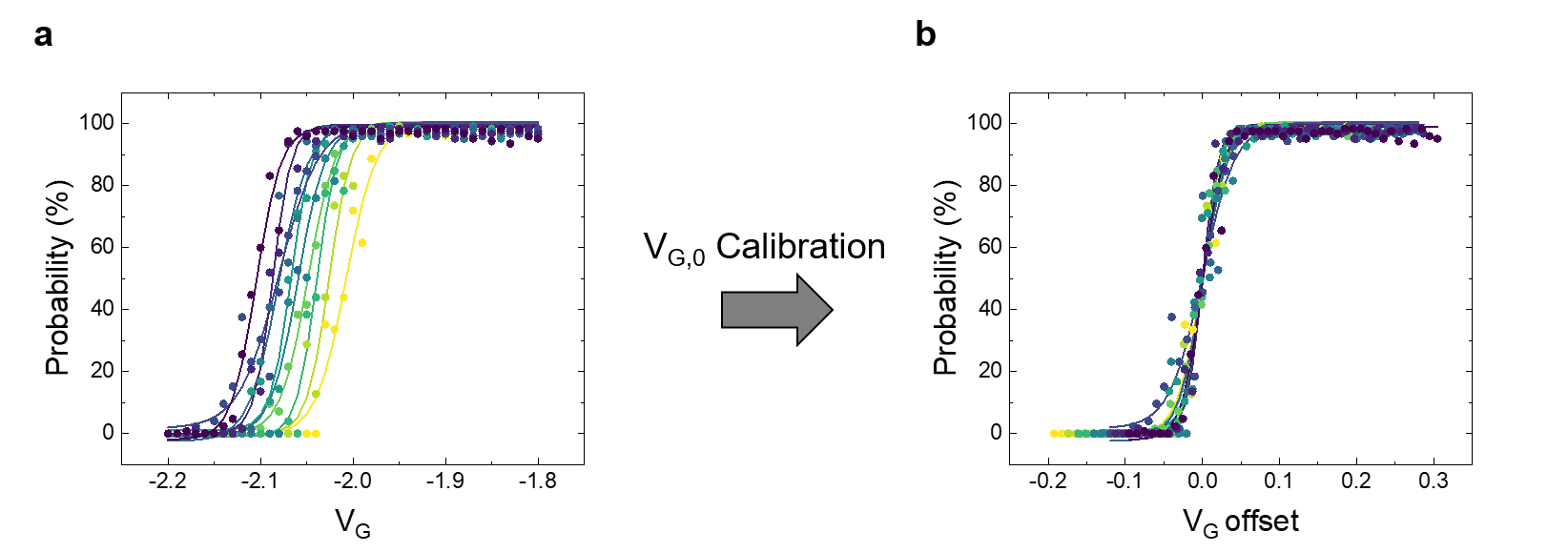


Figure S7 | Device-to-device variation control through *V*_G_ modulation.

(a) The probability of STL versus *V*_G_ measured in ten devices with fixed *V*_D_ = 3.3 V. (b) The probability of STL versus *V*_G_ after *V*_G,0_ calibrations. *V*_G,0_ is the mean value of *V*_G_ at which the probability of STL of ten devices is 50 %.


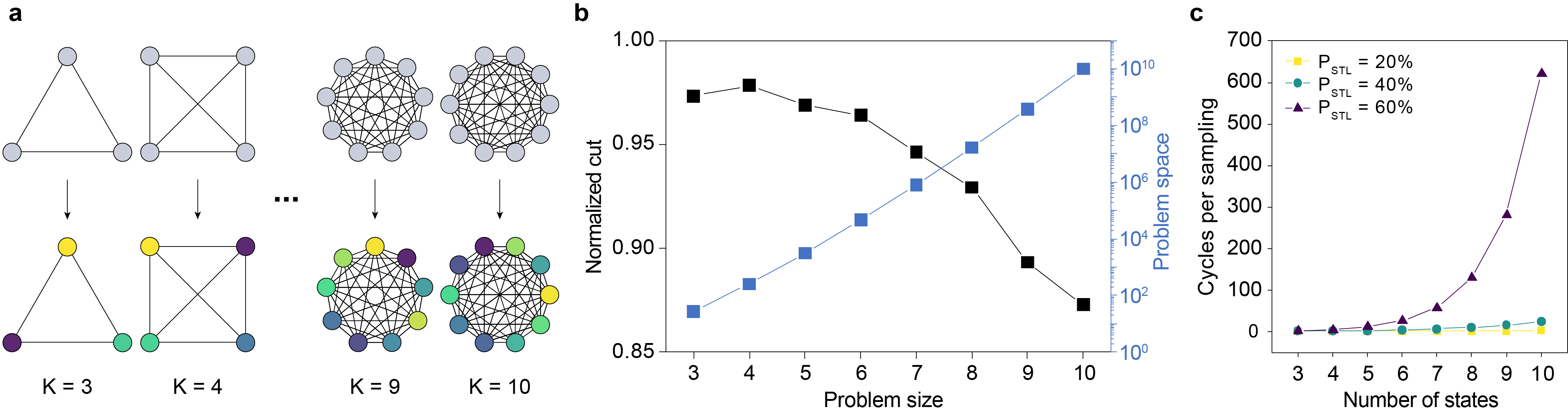


Figure S8 | Experimental results of solving max-K-cut problems.

Max-K-cut problems of fully connected graphs with *K* nodes were solved. Instead of the one-hot sampling suggested in the main text, which uses a single cycle, 25 cycles were used to sample the one-hot state (*N*_cyc_ of 25). (a) Fully connected graphs with *K* nodes for max-K-cut problems. (b) Normalized cut and problem space for different fully connected graph sizes. (c) Cycles per sampling required for one-hot sampling, depending on the number of states. The results show that extensive cycles, compared to the suggested one-hot sampling in the main text (*N*_cyc_ = 1, Figure 4h), decrease the normalized cut value for larger *K*, even though the problem has a simple solution (coloring all the nodes differently). It is attributed to synchronous update, where the nodes are simultaneously updated, inhibiting random sampling from the problem space. Specifically, *N*_cyc_ of 25 leads to a simultaneous update of all *K* nodes, which prevents the system from visiting all possible combinations, especially the answer where all the nodes are colored differently.

Figure S9 | Success probability of experimental max-K-cut problems.

This figure shows the success probabilities for experimentally solving fully connected graphs with various numbers of p-bit states. Each graph with *K* states has *K* nodes, as shown in Figure S8. As the maximum number of sampling cycles was set to 25 and the problem space increased exponentially, the success probability decreased according to the number of states. The previous state was maintained if the one-hot sampling failed in 25 cycles.


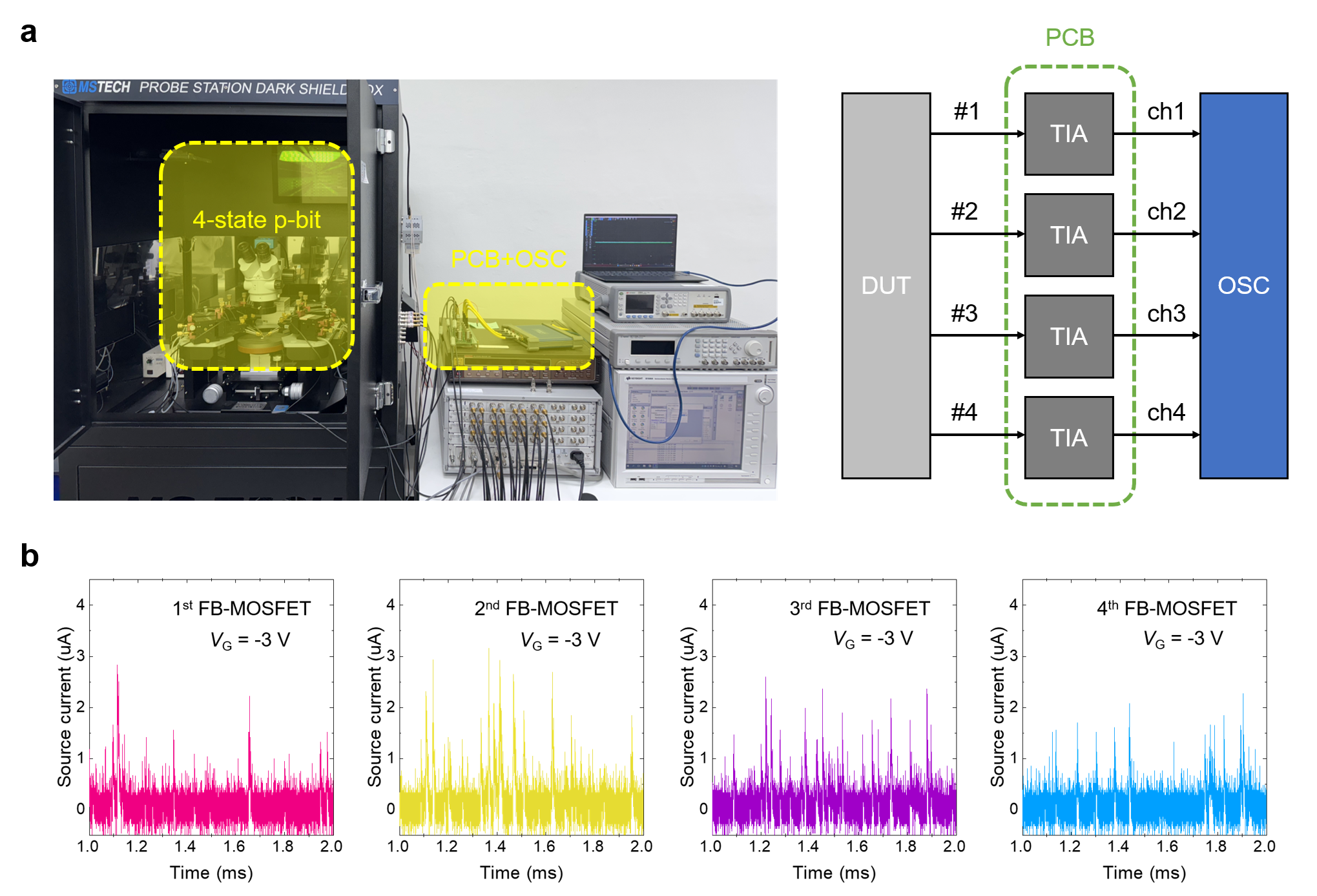


Figure S10 | The 4-state p-bit operation at 1 MHz.

(a) The optimized measurement setup. A custom PCB with enhanced grounding techniques was used in a shielded probe station to minimize noise and parasitic capacitance, enabling 1 MHz characterization. (b) Experimental demonstration of 1 MHz operation for a 4-state p-bit unit. The time-domain source current traces of each FB-MOSFET show distinct peaks operating at 1 MHz, verifying the device's capability for high-speed stochastic switching.


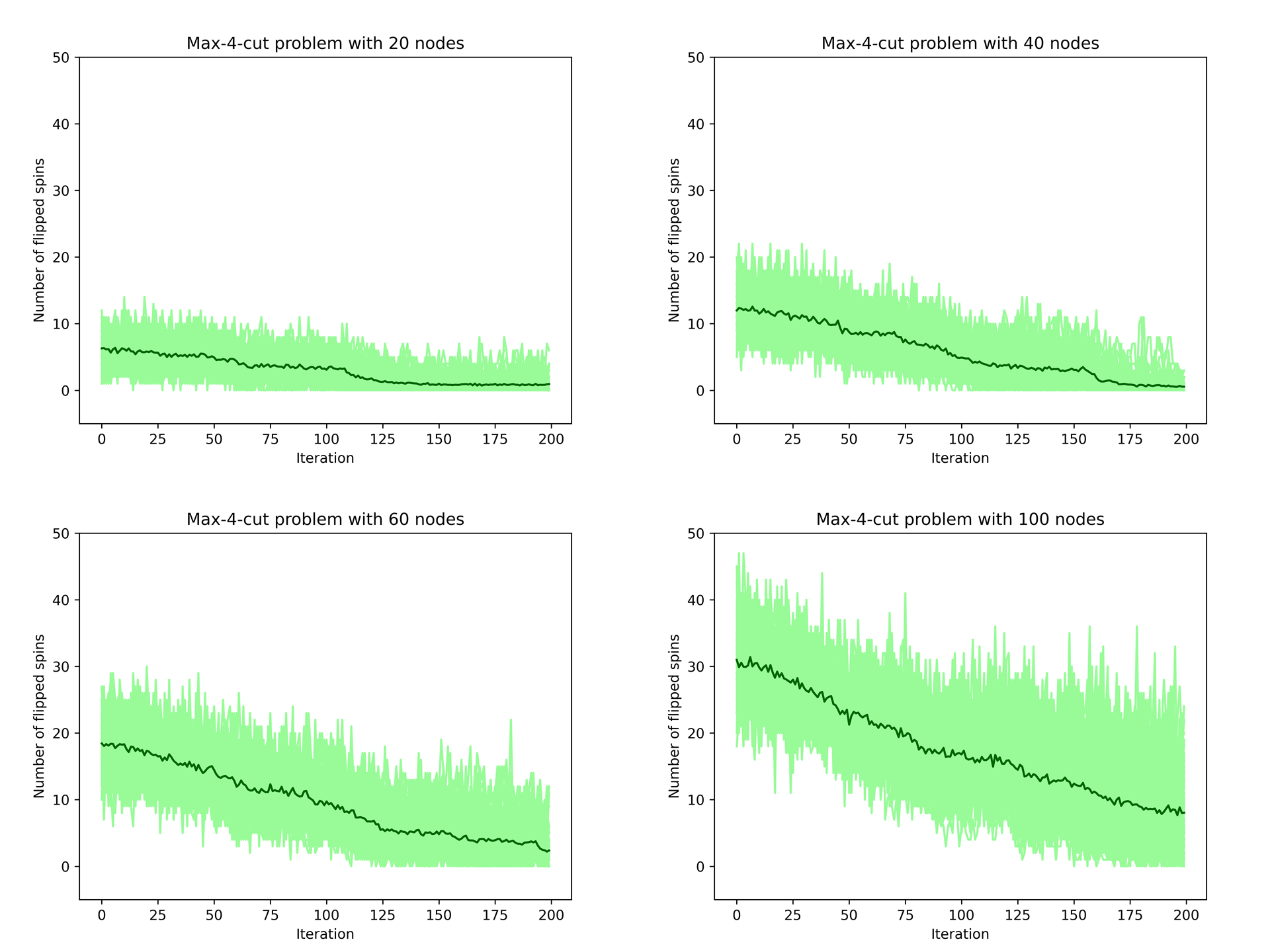


Figure S11 | Number of flipped states in solving max-4-cut problems.

The number of state flips in each iteration is shown for solving max-4-cut problems with different sizes. The number of flips is constrained even in early iterations, as the suggested one-hot sampling uses a single cycle for state updates. As the updated nodes are selected by an inherently random process, scalable annealing profiles, where the number of flipped spins decreases through iterations even for the large problem size (100), are generated regardless of the problem size. Most importantly, it enables parallel updates of the nodes, which eliminates the additional burden of asynchronous updates and reduces the sampling latency. Each result was obtained by solving a problem from the problem set 100 times.


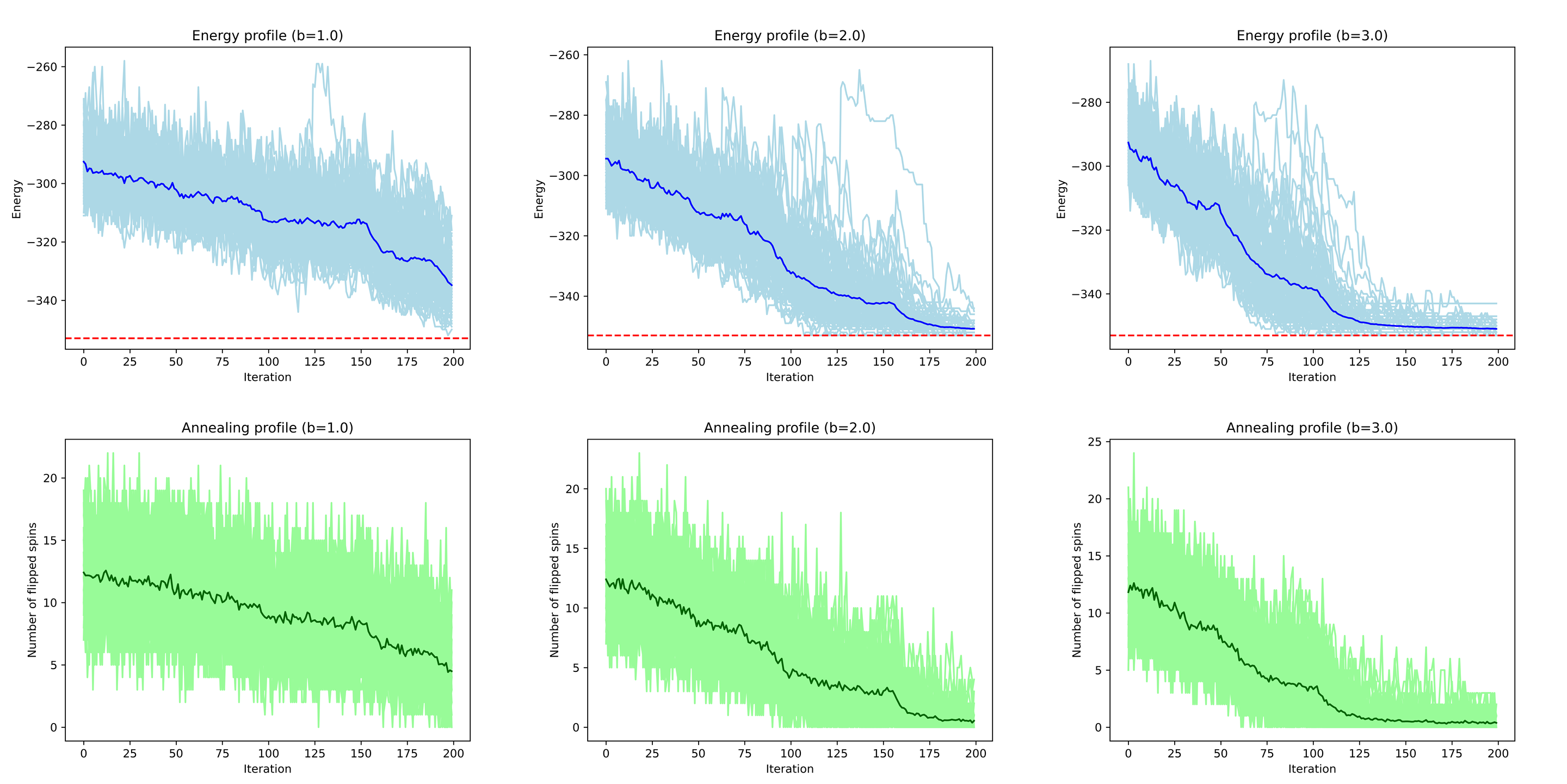


Figure S12 | Influence of parameter *b* on annealing.

A max-4-cut problem with 40 nodes was solved 100 times with different values of *b*. The strength of the feedback is linearly increased from 0.1 to the values specified in each figure throughout the iterations. The upper panel displays the energy profile, while the lower panel shows the annealing profile, which represents the number of flips per iteration. As the feedback strength increases, the system converges more quickly with fewer iterations. An adequate value of *b* was selected in the experiment and simulation based on the problem size and the number of iterations.

**
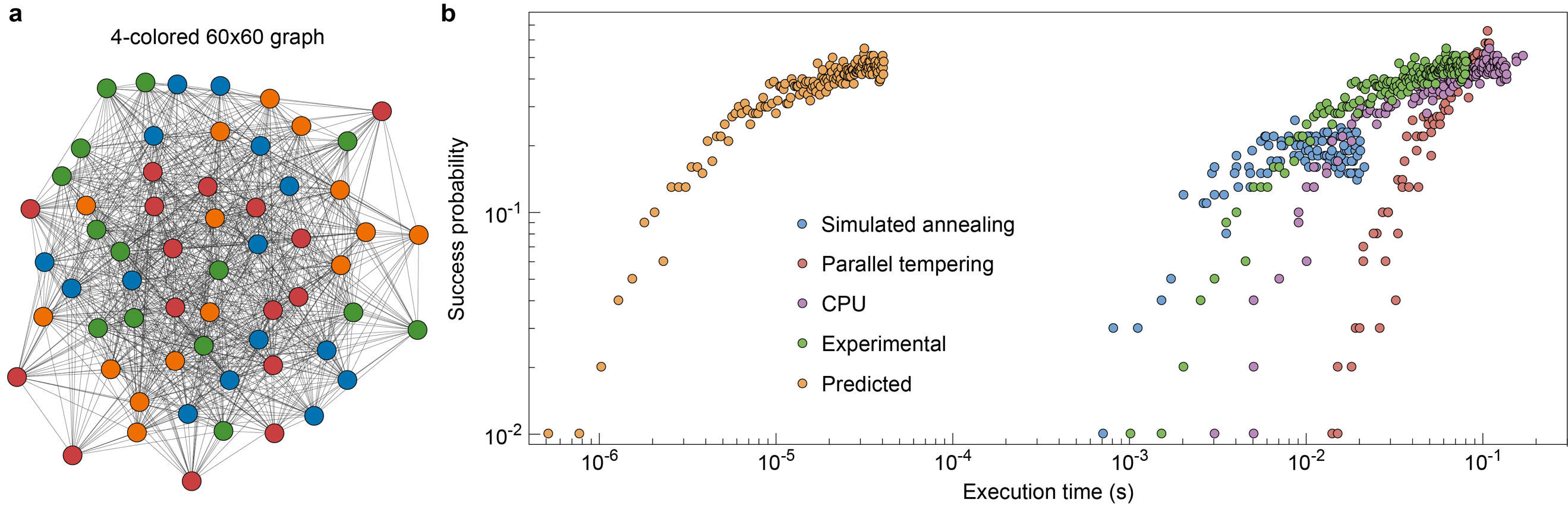
**

Figure S13 | Comparison of various systems to solve a max-4-cut problem.

(a) An example problem with 60 nodes. (b) The success probability of various systems with execution time. The max-4-cut problem of the graph in (a) was solved 100 times for each algorithm. The plots labeled 'CPU,' 'Experimental,' and 'Predicted' correspond to executing the suggested algorithm using a CPU, a 50 kHz clock frequency for multi-state p-bit units, and a 100 MHz clock frequency for multi-state p-bit units, respectively.


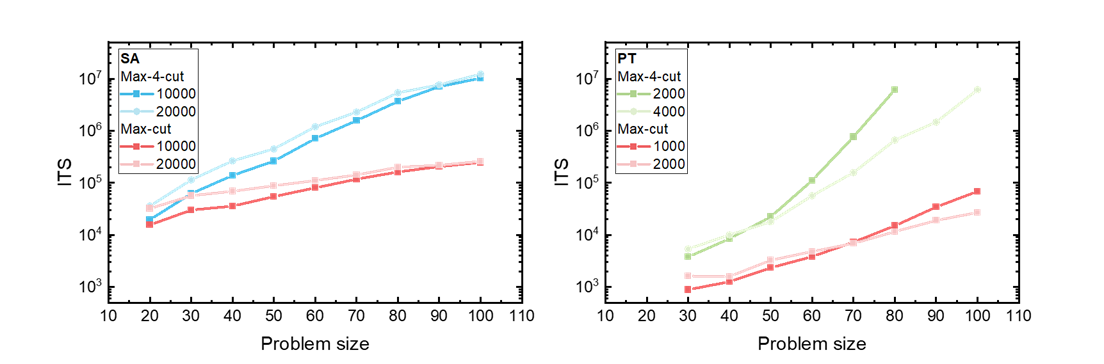


Figure S14 | Iteration-to-solution (ITS) of simulated annealing (SA) and parallel tempering (PT) on max-cut problems

The ITS on max-cut problems and max-4-cut problems are compared. Due to the difference in problem space (2*N* and 4*N*), ITS for 2*N*-node max-cut problems yields similar values to those of *N*-node max-4-cut problems. The same problem set was used for max-cut problems, which consist of 20 instances per problem size, solved 100 times each.

Figure S15 | Comparison of the one-hot sampling methods on max-4-cut problems

This figure shows the ITSs to solve the max-4-cut problems for various *N*_cyc_s. Increased *N*_cyc_ leads to two problems: longer latency and non-random sampling. Initially, for small problem sizes, higher *N*_cyc_ results in a linear increase in iterations due to increased sampling latency. Moreover, as the problem size increases, ITS increases to infinite, meaning the system cannot find a solution due to non-random sampling. For the ITS calculation, the problem set in Figure 4 was solved using 1000 iterations (*N*_cyc_ × 1000).

Table S1 | Energy consumption for solving COPs.

| Elements | Energy (pJ) | Count | Configuration |
| --- | --- | --- | --- |
| FB-MOSFET array | 0.725 | 1 | 50 x 4-states |
| DAC | 0.048 | 250 | Gate and drain voltage |
| Comparator | 0.110 | 200 | Source readout |
| MUX + decoder | 0.048 | 200 | Gate (200) and drain (1) |
| I/O register (2 KB) | 1.488 | 1 | *V*_G_ control buffer |
| Total | 45.81 |  |  |

Energy consumption to solve max-4-cut problems of 50 nodes was calculated based on Neurosim^[1]^ and previous work^[2–4]^, assuming the 16 nm technology node and device characteristics of FB-MOSFET with the clock frequency of 1 GHz. Fully parallel operation was assumed in the energy calculation.

Supporting Note 1 | Method for Calculating the Ideal Probability for 35 Combinations.

The tunability of the multi-state p-bit unit is verified by analyzing the cases where different *V*_G_ values are applied to each FB-MOSFET. By quantizing the *V*_G_ applied to each FB-MOSFET in the 4-state p-bit unit, 35 combinations could be obtained. The 35 combinations include all possible configurations of a 4-state node, with each state assigned one of the quantized probabilities: 0.2, 0.4, 0.6, or 0.8. Specifically, the number of combinations is calculated from _4_H_4_.

For example, if the *P*_STL_ of devices #1 to #4 is 20, 40, 60, and 80 %, by selecting the *V*_G_ values of -2.2, -2.15, -2.1, and -2.05 V for each device, respectively, the output probability of [1000], [0100], [0010], and [0001] vectors for one cycle is ideally 0.96, 2.56, 5.76, and 15.36 %. The ideal value can be obtained by calculating the probability that only one device is turned on for one cycle, which is approximately 0.96 % for the [1000] vector. This is derived from 0.2ⅹ(1-0.4)ⅹ(1-0.6)ⅹ(1-0.8). For all 35 possible combinations, the average of 100 results for each case is calculated.

Supporting Note 2 | Transition from a Complex COP (max-4-cut) to Max-Cut.

The transition from max-4-cut to max-cut based on binary p-bits was performed using the duplication shown in Figure S5. After duplicating each node into binary nodes to represent four colors in the max-4-cut problem, the connectivity is transferred to duplicated nodes representing the same colors. Then, all nodes are connected to the constraint node to ensure a valid solution. By setting the constraint node as +1, four nodes are more likely to have three -1 states and one +1 state. A valid solution is a set of nodes that meet this constraint, where each duplicate node with a unique state is selected as the state of the corresponding combined node. For example, if one of the duplicated nodes representing the color 'purple' is in state +1 and others are in state -1, the state of the combined node is 'purple'. However, if more than one node is in the same state as the constraint node, it results in an invalid solution.

Then, this transitioned max-cut problem was mapped into the Ising model for optimization. The strength of two connections, one between duplicated nodes and the other between a node and a constraint, can be optimized to solve the problem. However, this optimization can cause a trade-off between validity and success probability, as demonstrated in previous works.^[2]^ Moreover, the problem space increases exponentially from 4^N^ to 2^4N+1^, deteriorating the system's performance.

Supporting Note 3 | Overview of Ising Model and Annealing in Binary Probabilistic Computing

The Ising model, initially used in statistical physics, is now frequently applied to represent binary variables for a COP. In this representation, each binary variable is encoded as a spin, $s_{i} \in\{-1, 1\}$ corresponding to '0' and '1' in the computational domain. The energy function of the Ising model is typically expressed as Eq. (S1).

| $E=-\left( \sum_{i,j} J_{ij}s_{i}s_{j}+ \sum_{i} h_{i}s_{i} \right)$ | (S1) |
| --- | --- |

, where $J_{ij}$ is the coupling strength between spins i and j, and $h_{i}$ is the external magnetic field acting on spin i. To analyze how each $s_{i}$ contributes to the total energy, the partial derivative of $E$ for $s_{i}$ is calculated as Eq. (S2).

| $-\frac{\partial E}{\partial s_{i}}=\sum_{j} J_{ij}s_{j}+h_{i}$ | (S2) |
| --- | --- |

The problem is then solved by updating the spin states to minimize the total energy through these calculation results. However, because COPs have a complex energy landscape, the system is often prone to becoming stuck in local energy minima, a tendency that becomes more severe in systems consisting of deterministic bits. However, it is possible to escape the local minima and reach the global minimum in systems composed of p-bits, where the probability of '0' and '1' states can be controlled.

To guide this stochastic process toward convergence, an annealing schedule is employed. Annealing is a process inspired by physical annealing of materials, in which a system is slowly cooled to reach a global energy minimum.^[5,6]^ In COPs, annealing helps the system escape local minima and reach the global minimum. During the early iterations at high temperatures, the states of the p-bit actively fluctuate as they seek lower energy levels. However, state changes are suppressed in the later iterations at lower temperatures, and the current state remains.^[7,8]^ This process can be obtained by multiplying the inverse temperature $\beta$ to the calculated value from Eq. (S2). Following the annealing schedule, it changes from low values to high values linearly or nonlinearly over time.

Supporting Note 4 | Detailed Sampling Method Using a Custom Board.

This work utilizes the sequential sampling process, where a single node is sampled by applying *V*_in_ to the common drain and arbitrarily selecting *V*_G_ values in each p-bit. Then, the same sampling process is conducted *N* times for a problem with *N* nodes corresponding to a single iteration. It should be noted that sampling for all *N* nodes can be performed in parallel on the integrated chip; however, this work adopted a sequential process due to the limited capacity of the custom board. When one iteration is complete, *N* one-hot vectors are obtained, as described in Section 2.2, which are used to update the *V*_G_ values in each p-bit during the subsequent iteration. The subsequent section describes the detailed *V*_G_ update method for the max-4-cut problem.

Supporting Note 5 | Gate Voltage Feedback Calculation in Max-K-Cut Problem.

Similar to the conventional approach in probabilistic computing, input proportional to the derivative of the target energy function is applied to each multi-state p-bit unit. In contrast to two-terminal p-bits, the calculated input was applied as *V*_G_ in this work. Since a given *V*_G_ determines a specific *P*_STL_ at the common *V*_D_, the feedback in this note is expressed as probability rather than voltage. In the experiment, *V*_G_ corresponding to a particular probability was selected from a look-up table based on previously measured data.

| $-\frac{\partial E}{\partial\vec{s}_{i}}=-\sum_{j}^{N} A_{i,j}\vec{s}_{j}$ | (S3) |
| --- | --- |

After sampling all the p-bits in every iteration, a probability update proportional to the derivative value in Eq. (S3) was calculated. The vector of gradient counts the states of p-bits connected to the selected p-bit, $\vec{s}_{i}$. For example, the gradient vector [-x_1_, -x_2_, -x_3_, -x_4_] in the max-4-cut problem corresponded to x_1_, x_2_, x_3_ and x_4_ connected p-bits, each with state [1, 0, 0, 0], [0, 1, 0, 0], [0, 0, 1, 0] and [0, 0, 0, 1], respectively. While the gradient vector could guide the states to converge by indicating the connected states, negative elements would decrease the probabilities across all entries and add extra complexity to the sampling process. The currently sampled states were then used as the baseline to adjust the gradient vector. For instance, the gradient vector was adjusted to [0, x_1_-x_2_, x_1_-x_3,_ x_1_-x_4_] if the current state was [1, 0, 0, 0]. Eq. (S4) shows the probability update, where $\vec{e}$ and $\vec{P}_{\vec{s}_{i}}$ refer to a vector of ones ([1, 1, 1, 1]) and *P*_STL_vector for state $\vec{s}_{i}$, respectively.

| $\Delta\vec{P}_{\vec{s}_{i}}=\sum_{j}^{N} A_{i,j}((\vec{e}-\vec{s}_{i})\left( \vec{s}_{j}\cdot\vec{s}_{i} \right)-\vec{s}_{j}(1-\left( \vec{s}_{j}\cdot\vec{s}_{i} \right)))$ | (S4) |
| --- | --- |

Then, the gradient vector was scaled and accumulated to the initial probability, $\vec{P}_{initial}$, where all the entries are set as 0.3 for the max-4-cut problem. The strength of the feedback was determined by the constant multiplier *b*, which was linearly increased to reduce noise and guide the system toward the global minimum in subsequent iterations. However, it was set as a constant for the Boltzmann sampling in Figure 4. Eq. (S5) describes accumulation.

|  |  |
| --- | --- |
| $\vec{P}_{\vec{s}_{i}}= \vec{P}_{initial}+b\Delta\vec{P}_{\vec{s}_{i}}$ | (S5) |

For the experimental demonstration, the parameter *b* was increased linearly from 0.1 to 2.0 ~ 5.0, depending on the problem size. Note that the calculated updating amounts in Eq. (S4) were first normalized with respect to the problem size. Specifically, it was divided by the square root of the problem size to fine-tune *b*. The initial probabilities for each multi-state p-bit unit were set to 0.3 and modulated after each iteration. In later iterations, as the b value increased, most nodes tended to remain in their previous states. The accumulation resulted in fewer nodes to flip, where a specific state dominates. The quantized probabilities (10 states), $\vec{P}_{\vec{s}_{i}}$, were translated into *V*_G_ through a look-up table, with the calibration process discussed in Figures S2 and S7.

All feedback probabilities and controls, including the lookup table, were calculated in the MCU using Python code. First, the multi-state p-bits are sampled by the ADC after pulses are injected through the DAC. Then, the feedback probabilities in Eqs. (S1) ~ (S3) are calculated by the MCU, which implements the coupling of p-bits (adjacency). Lastly, the *V*_G_ of the FB-MOSFET is determined by the pre-measured devices' firing properties, as shown in the firing probability-voltage characteristics in Figure 1d.

Supporting Note 6 | Calibration of Multiple FB-MOSFETs.

The calibration of 10 FB-MOSFETs as p-bits was performed in two steps. In the first step, the *P*_STL_ versus *V*_G_ data for each device were fitted to a sigmoidal equation (S6), resulting in individual curves for each device.

| $P_{STL,fitted}=\frac{1}{1+ e^{a\times(V_{G}-b)}}$ | (S6) |
| --- | --- |

, where *a* and *b* represent the slope and shift, respectively. It was observed that the sigmoidal curves of the 10 devices exhibited variations primarily along the x-axis, while the slope remained nearly consistent (left panel of Figure S7).

Then, the mean value of the x-axis shift across all 10 devices (*V*_G,0_) was extracted. This value was then used as a reference, enabling a consistent offset adjustment for each device. As a result, all devices were aligned to a common input range, ensuring uniformity in the system (right panel of Figure S7).

It should be noted that this x-axis offset calibration was sufficient to maintain effective performance, given the fault-tolerant nature of stochastic systems. The precision requirements were relatively relaxed because the p-bits inherently tolerate minor variations in probability values. By adopting this method, the calibration of the p-bits was efficiently managed, ensuring consistent and predictable behavior in subsequent probabilistic computing.

Supporting Note 7 | Comparison of various systems to solve a max-4-cut problem.

A max-4-cut problem with 60 nodes, as shown in Figure S12a, was solved using various algorithms to evaluate scalability and predict performance improvements in an integrated chip. Figure S12b shows that the suggested algorithm, when combined with the experimental setup (50 kHz clock frequency, green dots), performs similarly to the CPU (purple dots) and parallel tempering (red dots). Although simulated annealing (blue dots) performs better with a shorter execution time, it fails to scale well with more iterations (longer execution time). The predicted curve on the leftmost of Figure S12b assumed a 100 MHz clock frequency in the experiment, which could not be achieved due to parasitic effects in the fabricated PCB. However, it demonstrates the capability of the suggested method to significantly decrease execution time when integrating all the circuits into the chip. This projection is grounded in the nanosecond-scale stochastic switching speed of FB-MOSFETs previously reported, whereas the measured performance in the present work is limited by control and data-acquisition bottlenecks on the proof-of-concept board. Realizing this potential hardware speedup will further require addressing circuit-level challenges, including synchronization, timing jitter, parasitic RC delay, and thermal management under high-frequency operation.

Supporting Note 8 | Performance Comparison in Table 1.

Table 1 presents a comparison between the present Potts machine and various Ising machines in terms of solved problems, time-to-solution (TTS), and energy-to-solution (ETS). For works other than this one and for parallel tempering on the CPU, the max-cut problem with 100 nodes and density 0.5 was solved. In contrast, this work and parallel tempering on the CPU solved a max-4-cut problem with the Potts model description. For the first column (this work), a clock frequency of 1 GHz, which is the STL limit demonstrated so far, was assumed. TTS was obtained from Figure 5, where 20 problems with 50 nodes and a density of 0.5 were solved.

Here, 50 nodes were selected by simply comparing the problem space of the max-4-cut (4^N^) and the max-cut problem (2^N^). For instance, the previous work implemented simulated annealing for max-cut problems and presented ITS as a performance metric.^[9]^ For the same problem space (4^N^), it showed a similar but slightly lower ITS (max-cut problem with 2N nodes) to the ITS in this work (max-4-cut problem with *N* nodes). This approach was further validated by simulated annealing for the Potts model with *q* = 2 (Figure S13). The result is similar to or slightly lower than that of the previous work, justifying the calculation.

For the parallel tempering, a similar approach was adopted. In addition to the result from Figure 5, parallel calculation of 20 replicas in the algorithm was assumed, further decreasing TTS. We also assumed a power consumption of 5 W in the CPU core. The obtained TTS of 9 ms showed a similar order of magnitude compared to previous work that solved a max-cut problem with the same problem space using the parallel tempering algorithm on an Intel Xeon CPU E5-1650 v2.^[10]^

Supporting references

[1] Y. Luo, X. Peng, S. Yu, in *ACM International Conference Proceeding Series*, **2019**.

[2] J. Si, S. Yang, Y. Cen, J. Chen, Y. Huang, Z. Yao, D. J. Kim, K. Cai, J. Yoo, X. Fong, H. Yang, *Nat. Commun.* **2024**, *15*, DOI 10.1038/s41467-024-47818-z.

[3] F. Cai, S. Kumar, T. Van Vaerenbergh, X. Sheng, R. Liu, C. Li, Z. Liu, M. Foltin, S. Yu, Q. Xia, J. J. Yang, R. Beausoleil, W. D. Lu, J. P. Strachan, *Nat. Electron*. **2020**, *3*, DOI 10.1038/s41928-020-0436-6.

[4] S. in Yi, J. D. Kendall, R. S. Williams, S. Kumar, *Nat. Electron.* **2023**, *6*, DOI 10.1038/s41928-022-00869-w.

[5] P. Hauke, H. G. Katzgraber, W. Lechner, H. Nishimori, W. D. Oliver, *Reports on Progress in Physics* **2020**, *83*, DOI 10.1088/1361-6633/ab85b8.

[6] L. Ingber, *Math. Comput. Model* **1993**, *18*, DOI 10.1016/0895-7177(93)90204-C.

[7] L. Chen, K. Aihara, *Neural Networks* **1995**, *8*, DOI 10.1016/0893-6080(95)00033-V.

[8] Y. He, *IEEE Trans. Neural Netw.* **2002**, *13*, DOI 10.1109/TNN.2002.804314.

[9] M. Jiang, K. Shan, C. He, C. Li, *Nat. Commun.* **2023**, *14*, DOI 10.1038/s41467-023-41647-2.

[10] R. Hamerly, T. Inagaki, P. L. McMahon, D. Venturelli, A. Marandi, T. Onodera, E. Ng, C. Langrock, K. Inaba, T. Honjo, K. Enbutsu, T. Umeki, R. Kasahara, S. Utsunomiya, S. Kako, K. I. Kawarabayashi, R. L. Byer, M. M. Fejer, H. Mabuchi, D. Englund, E. Rieffel, H. Takesue, Y. Yamamoto, *Sci. Adv.* **2019**, *5*, DOI 10.1126/sciadv.aau0823.
